# Prolonged running reduces speed at the moderate-to-heavy intensity transition without additional reductions due to increased eccentric load

**DOI:** 10.1007/s00421-025-05792-4

**Published:** 2025-04-29

**Authors:** Andrew M. S. Barrett, Ed Maunder

**Affiliations:** 1 Sports Performance Research Institute New Zealand, Auckland University of Technology, Auckland, New Zealand; 2 School of Cardiovascular and Metabolic Health, College of Medical, Veterinary and Life Sciences, University of Glasgow, Glasgow, UK

**Keywords:** Running, Thresholds, Durability, Eccentric, Endurance, Marathon

## Abstract

**Aim:**

To investigate the impact of prolonged running on speed at the moderate-to-heavy intensity transition, and whether increased eccentric load modifies exercise-induced shifts in the moderate-to-heavy intensity transition.

**Methods:**

Twelve endurance-trained runners (4 females, 8 males, peak oxygen uptake 51.5 mL kg^−1^ min^−1^ in females and 56.1 mL kg^−1^ min^−1^ in males) participated. Following trials to assess speed at the first ventilatory threshold (VT_1_) on a 0% gradient and -10% gradient, participants completed two trials: (i) level trial (LVL): 120-min of running at 0% gradient and (ii) downhill trial (DH): 120-min of running with 90-min at 0% gradient and 6 × 5-min intervals at -10% gradient (DH). Running was conducted at 90% of VT_1_ speed for respective gradients. Speed at VT_1_ on 0% gradient, perceived leg muscle soreness, and maximal voluntary isometric knee extensor torque were assessed pre- and post-prolonged exercise.

**Results:**

An effect of time was observed for speed at VT_1_ (∆-6.2 ± 3.6% in LVL and ∆-7.6 ± 3.2% in DH, *P* < 0.001), with no effect of condition (LVL vs. DH,* P* = 0.382), or time × condition interaction (*P* = 0.295). Reduced metabolic energy expenditure (metabolic power) significantly contributed to the reduced speed at VT_1_ in both trials (*P* < 0.001), whereas changes in running economy (energetic efficiency) did not (*P* = 0.228).

**Conclusion:**

Prolonged moderate-intensity running reduced speed at the moderate-to-heavy intensity transition, but this was not exacerbated by increased eccentric load. Reduced speed at the moderate-to-heavy intensity transition following prolonged running was primarily attributed to decreased metabolic power.

**Supplementary Information:**

The online version contains supplementary material available at 10.1007/s00421-025-05792-4.

## Introduction

The physiological response to exercise can be divided into three distinct intensity domains (Jones et al. 2019). During moderate-intensity exercise, disturbances to muscle metabolic and ionic homeostasis are minimal, and blood lactate concentrations remain close to baseline. During heavy-intensity exercise, a delayed steady state is established for blood lactate concentration, whole-body oxygen consumption ($\dot{V}O_{2}$), and muscle metabolic and ionic homeostasis (e.g., PCr, Pi, H^+^). In contrast, during severe-intensity exercise, no steady state is attained, and these variables progress to a peak or nadir at task failure (Jones et al. 2008; Burnley et al. 2010; Black et al. 2017). Prolonged cycling reduces the power output at the intensity domain transitions, with a non-linear time course and considerable inter-individual variability (Clark et al. 2018; Clark et al. 2019a, b; Stevenson et al. 2022; Gallo et al. 2024; Hamilton et al. 2024). Resilience to prolonged exercise-induced degradation of intensity domain transitions has been termed ‘durability’ (Maunder et al. 2021) and has been proposed as a key performance determinant (Maunder et al. 2021; Jones 2023).

The effects of prolonged exercise on intensity domain transitions have only been investigated in cycling thus far, with limited research in running (Nuuttila et al. 2024). Key differences between running and cycling that could influence durability include use of the stretch–shortening cycle and the induction of muscle damage (Jones 2023), and greater loss of neural function, during running (Brownstein et al. 2022). The stretch–shortening cycle is characterised by repetitive eccentric and concentric muscle activity (Bijker et al. 2002). Eccentric activity, which is largely absent in cycling, is associated with significantly greater mechanical loads than concentric contractions (Marcora and Bosio 2007). The resultant mechanical stress can damage contractile proteins (McCully and Faulkner 1986), disrupt myofilament structure (Morgan 1990; Talbot and Morgan 1996; Proske and Morgan 2001), and damage the dystrophin complex (Gao and McNally 2015; Owens et al. 2019). This structural damage can impair the function of contractile apparatus, excitation–contraction coupling and glucose oxidative function within individual muscle fibres (Macpherson et al. 1996; Tee et al. 2007). A reduction in speed at the moderate-to-heavy intensity transition during prolonged running may be attributed to two broad factors: reduced metabolic energy expenditure before transitioning to heavy intensity responses (‘metabolic power’, i.e. lower kcal^.^min^−1^ at the transition) and reduced running economy (‘energetic efficiency’, i.e. greater kcal^.^min^−1^ at a given speed; Stevenson et al. 2022). It is therefore plausible that the mechanical stress and muscle damage during running might impair contractile function in individual muscle fibres, reduce the size of the active fibre pool, and therefore contribute to a decline in speed at the moderate-to-heavy intensity transition via effects on metabolic power and energetic efficiency. Negatively-graded running intensifies the eccentric phase of the stretch–shortening cycle (Bontemps et al. 2020), resulting in increased markers of exercise-induced muscle damage, such as perceived muscle soreness and reduced maximal voluntary force production (Eston et al. 1996, 2000; Rowlands et al. 2001; Braun and Dutto 2003). Therefore, performing relative intensity-matched running on level and negative gradients may offer an ecologically valid model for studying the impact of eccentric load on the moderate-to-heavy intensity transition in running.

Muscle glycogen depletion might also contribute to a loss of metabolic power by impairing the contractile function of individual fibres, as glycogen depletion beyond a threshold may render individual fibres inexcitable (Cairns and Renaud 2023; Ortenblad et al. 2013; Nielsen et al. 2024). This is pertinent in the prolonged exercise context, as the most-oxidative, type I fibres are preferentially activated, and therefore most rapidly glycogen-depleted, meaning the active fibre pool may be progressively less oxidative as exercise progresses (Nielsen et al. 2024). A less-oxidative fibre pool may plausibly exhibit a heavy-intensity response at a lower metabolic energy expenditure and running speed. Greater use of type II fibres is also likely to reduce energetic efficiency (Swinnen et al. 2024). Also, some studies have observed reduced running economy over time during moderate/heavy intensity running (Unhjem 2024; Zanini et al. 2024). This could similarly be a result of a shift in the exercising fibre pool from oxidative type I to less oxidative type II fibres (Jones et al. 2011; Nielsen et al. 2024) and contribute to the reduction in speed at the moderate-to-heavy intensity transition via effects on energetic efficiency. Therefore, further investigation of the effect of prolonged running on speed at the moderate-to-heavy intensity transition is warranted.

Therefore, the aims of this study were to investigate: (i) the effect of prolonged running on speed at the moderate-to-heavy intensity transition, (ii) the contribution made by metabolic power and energetic efficiency to prolonged running-induced changes in speed at the moderate-to-heavy intensity transition, and (iii) whether increasing the eccentric load of running while remaining in the same intensity domain affects the magnitude of change in speed at the moderate-to-heavy intensity transition. We hypothesised that: (i) prolonged running would decrease speed at the moderate-to-heavy transition, (ii) reduced energetic efficiency and metabolic power would contribute to reduced speed at the moderate-to-heavy intensity transition, and (iii) greater eccentric load would exacerbate the reduction in speed at the moderate-to-heavy intensity transition.

## Methods

### Ethical approval

This study adhered to the Declaration of Helsinki, 2013. The Auckland University of Technology Ethics Committee approved all procedures (23/362), and participants provided written informed consent prior to participation. This study was not registered in a database. Raw data are available upon request.

### Participants

Twelve endurance-trained runners participated in this investigation (Table 1). Participants were free from illness and musculoskeletal injury (> 3 months), had no history of cardiovascular disease, trained ≥ 3 times week^−1^, ran ≥ 30 km week^−1^, and could run 5 km in < 20-min for males and < 23-min for females (self-reported). Participants completed a general health screening questionnaire and provided written informed consent. Participants received a report detailing key data upon study completion.

**Table 1:** Participant characteristics

N = 12	Male (N = 8)	Female (N = 4)
Age (y)	39 ± 8	37 ± 9
Height (cm)	182.4 ± 6.6	163.0 ± 3.0
Mass (kg)	74.4 ± 11.2	59.0 ± 6.9
$\dot{V}O_{2} {\text{peak}}$(mL kg^−1^ min^−1^)	56.1 ± 3.5	51.5 ± 6.9
PFO (g min^−1^)	0.58 ± 0.10	0.47 ± 0.26
Training volume (km week^−1^)	79 ± 31	60 ± 22
Training frequency (sessions week^−1^)	6 ± 1	7 ± 2

Data are presented as mean ± standard deviation. *PFO* peak fat oxidation rate, $\dot{V}O_{2} {\text{peak}}$ peak rate of oxygen uptake

### Study design

**Fig. 1:**
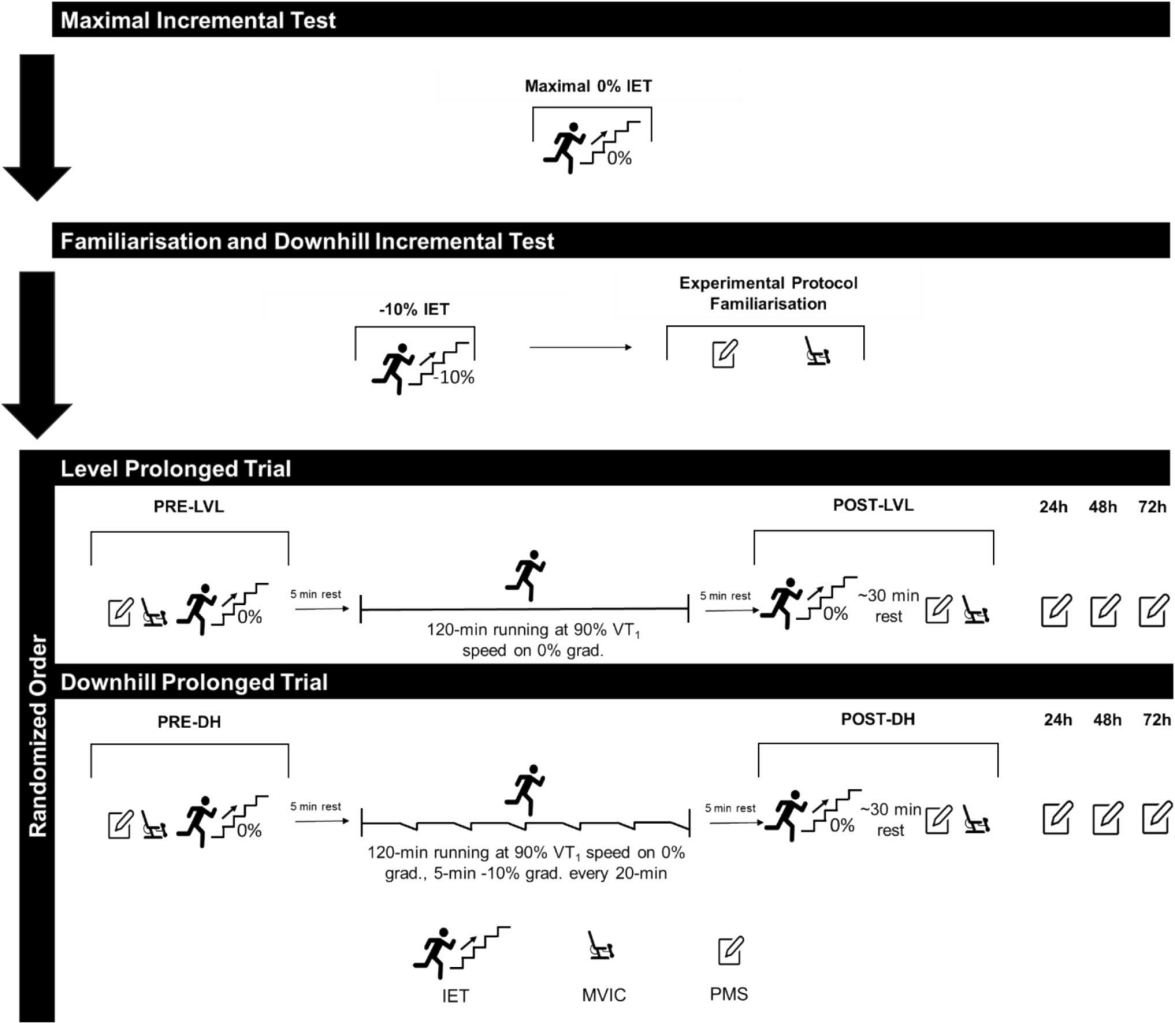
Schematic of study design *Grad*. gradient, *submax. *submaximal, *IET* incremental exercise test, *VT*_*1*_ first ventilatory threshold, *MVIC* maximal voluntary isometric contraction, *PMS* perceived muscle soreness

Participants visited the laboratory on four occasions. Visit 1 was a characterisation trial involving an incremental exercise test (IET) to estimate the running speed at VT_1_ for use in subsequent trials and peak rate of oxygen uptake ($\dot{V}O_{2} {\text{peak}}$; Fig. 1). Visit 2 included a submaximal IET on a − 10% gradient to determine ‘downhill’ running speed at VT_1_ and familiarisation with an exercise induced muscle damage indicators battery (Fig. 1). Visits 3 and 4, conducted in random counterbalanced order, began with the exercise induced muscle damage indicators battery to assess maximal voluntary isometric knee extensor torque and perceived leg muscle soreness, followed by a 0% gradient submaximal IET to assess speeds at VT_1_ (PRE-LVL and PRE-DH). Participants then completed either: (i) 120-min of running on a 0% gradient at 90% of ‘level’ VT_1_ speed estimated in visit 1 (LVL), or (ii) 120-min of running, with the last 5-min of every 20-min on a − 10% gradient at 90% of the ‘downhill’ VT_1_ speed estimated in visit 2, and the remainder on a 0% gradient at 90% of ‘level’ VT_1_ estimated in visit 1 (DH). Following this, participants performed the 0% gradient submaximal IET to determine speed at VT_1_ and the exercise induced muscle damage indicators battery to assess maximal voluntary isometric knee extensor torque and perceived leg muscle soreness (POST-LVL and POST-DH). Perceived leg muscle soreness was also recorded at ~ 24-, ~ 48- and ~ 72-h after visits 3 and 4 (Fig. 1). All trials commenced at ~ 6 am in a temperature-controlled laboratory (18–20 °C). Visits 1 and 2 were ~ 2–7 days apart, while 2, 3, and 4 were 7–14 days apart. Participants arrived fasted (overnight ~ 10-h) for visit 1 and consumed a standard breakfast of ~ 1 g^.^kg^−1^ carbohydrates before visits 2–4. The same pair of running shoes was worn for all trials.

### Visit 1: characterisation trial

Participants reported to the laboratory for the initial maximal IET having fasted overnight (~ 10-h), ingested 1–2 L of plain water, and refrained from caffeine and vigorous exercise for ~ 24-h (Fig. 1). After completing a health screening questionnaire and providing written informed consent, height and mass were measured. Running commenced on a motorised treadmill (gradient 0%; Saturn 250-75R, HP Cosmos, Germany) with a 5-min warm-up at 9 km h^−1^. The IET began at 10 km h^−1^, with speed increased by 1 km h^−1^ every 3 min. Expired gas and heart rate were collected continuously using indirect calorimetry (TrueOne 2400, ParvoMedics, UT, USA) and a chest-strap heart rate monitor (Polar Electro Oy, Kempele, Finland). When respiratory exchange ratio ≥ 1.0 and a decrease in end-tidal partial pressure of carbon dioxide was observed, speed was increased by 1 km h^−1^ each minute until volitional exhaustion. The $\dot{V}O_{2} {\text{peak}}$ was accepted as the highest 15-s average rate of oxygen uptake ($\dot{V}O_{2}$), and VT_1_ was identified at the breakpoint of the $\dot{V}O_{2}$ vs. ventilatory equivalent for oxygen ($\dot{V}_{{\text{E}}} \cdot \dot{V}{\text{O}}_{{2}}^{{ - {1}}}$) relationship and confirmed by the breakpoint in end-tidal oxygen partial pressure. This $\dot{V}O_{2}$ was converted to speed by linear regression of the $\dot{V}O_{2}$ vs. speed relationship, using the average $\dot{V}O_{2}$ for the final minute of each 3-min stage. A detailed example of this process is provided in Supplementary Material S1. Heart rate, $\dot{V}O_{2}$ and metabolic energy expenditure at VT_1_ were calculated by linear regression of the respective variables vs. speed (Eq. 1). Expired gas data from the final minute of every 3-min stage was also used to quantify whole-body rates of fat oxidation using standard equations and peak fat oxidation was taken as the greatest final minute fat oxidation value (Jeukendrup and Wallis 2005; Eq. 1).

### Visit 2: downhill incremental exercise test and familiarisation

Participants reported to the laboratory 3–7 days following the characterisation trial, having been asked to consume a breakfast containing ~ 1 g^.^kg^−1^ carbohydrate and 1–2 L plain water. Participants recorded their breakfast using a smartphone-based application which features food from Australia and New Zealand (Easy Diet Diary, https://xyris.com.au/products/easy-diet-diary), to allow for replication in subsequent visits. Participants completed a submaximal IET on a − 10% gradient to determine ‘downhill’ speed at VT_1_ and were familiarised with the exercise induced muscle damage indicators battery (Fig. 1). Negative gradients were achieved by reversing the direction of travel of the treadmill belt, converting what was a positive gradient in the forward direction to a negative gradient.

Participants initially acclimated to the negatively graded treadmill (~ 5-min walking and light running). After indicating readiness, a 4-min warm-up at 9 km h^−1^ (0% gradient) was completed before the treadmill was adjusted to a -10% gradient. The IET comprised five 3-min stages, starting at 4 km h^−1^ below 120% of the ‘level’ speed at VT_1_ determined at visit 1. Speed increased by 2 km h^−1^ per stage, with the third stage at 120% of the ‘level’ VT_1_ speed. This value of 120% of the ‘level’ VT_1_ speed was selected for the third stage based on our pilot work and previous studies indicating VT_1_ speed on a -10% gradient was ~ 15–30% greater than at a 0% gradient, in endurance-trained runners (Lemire et al. 2020). Expired gas and heart rate were continuously measured and speed, heart rate, $\dot{V}O_{2}$ and metabolic energy expenditure at VT_1_ were estimated using the methods described for visit 1. A detailed example of this process is provided in Supplementary Material S2. One participant completed only four stages, but a clear increase in $\dot{V}O_{2}$ vs. $\dot{V}_{{\text{E}}} \cdot \dot{V}{\text{O}}_{{2}}^{{ - {1}}}$ was observed before cessation, allowing speed at VT_1_ to be estimated. Following a short rest, for familiarisation purposes, participants completed the exercise induced muscle damage indicators battery.

### Visit 3 and 4: experimental trials

Participants reported to the laboratory for the first of two, randomised, counterbalanced order, prolonged trials ~ 7 days after visit 2, and completed the second prolonged trial ~ 7–14 days later. Before both trials, participants were asked to replicate their breakfast from visit 2 and consume 1–2 L plain water. Prolonged trials began with the exercise induced muscle damage indicators battery. Following this, participants performed a 4-min warm-up running at 9 km h^−1^ (0% gradient) followed by a five-stage 0% gradient IET to determine speed and metabolic energy expenditure at VT_1_ (Eq. 1). The IET began 2 km h^−1^ below the estimated ‘level’ VT_1_ speed from visit 1, and the speed increased by 1 km h^−1^ every 4 min. Expired gas was measured continuously and speed and metabolic energy expenditure at VT_1_ were estimated using the methods described for visit 1. A detailed example of this process is provided in Supplementary Material S3. Cadence was measured continuously and used to calculate stride length (Stryd power meter, Boulder, Colorado, USA).

Subsequently, participants walked for 5 min at 5 km h^−1^ before running for either (i) 120 min on a 0% gradient at 90% of the ‘level’ VT_1_ speed estimated in visit 1 (LVL) or (ii) 120 min with the last 5 min of every 20 min on a − 10% gradient at 90% of the ‘downhill’ VT_1_ speed estimated in visit 2, and the remainder on a 0% gradient at 90% of the ‘level’ VT_1_ speed estimated in visit 1 (DH; Fig. 1). Continuous heart rate and cadence data were collected, with expired gases sampled every 20 min for 10 min. This ensured data were obtained for both ‘level’ and ‘downhill’ sections in the DH trial. Two 3-min averages for expired gases, heart rate, and cadence were calculated for each collection period (e.g., for min 10–20, averages were obtained from min 12–15 and 17–20). The 3-min expired gas samples were used to calculate whole-body rates of metabolic energy expenditure, fat oxidation and carbohydrate oxidation using standard equations (Jeukendrup and Wallis 2005; Eq. 1). Total metabolic energy expenditure, carbohydrate oxidation, and fat oxidation over the 120 min were calculated using the area under the curve method. Throughout the trial participants consumed plain water ad libitum.

$$ {\text{Whole-body rate of metabolic energy expenditure }}\left( {{\text{kcal min}}^{{ - {1}}} } \right)\, = \,0.{55}0\, \times \,\dot{V}CO_{2} \, \times \, + \,{4}.{471}\, \times \,\dot{V}O_{2} $$

$$ {\text{Whole-body rate of fat oxidation }}\left( {{\text{g min}}^{{ - {1}}} } \right)\, = \,{1}.{695}\, \times \,\dot{V}O_{2} {-}{1}.{7}0{1}\, \times \,\dot{V}CO_{2} $$

1 $$ {\text{Whole-body rate of carbohydrate oxidation }}\left( {{\text{g min}}^{{ - {1}}} } \right)\, = \,{4}.{344}\, \times \, \dot{V}CO_{2} {-}{3}.0{61}\, \times \, \dot{V}O_{2} $$

where $\dot{V}CO_{2}$ = rate of oxygen uptake (L min^−1^) and $\dot{V}CO_{2}$ = rate of carbon dioxide production (L min^−1^).

Following the 120-min prolonged run phase, participants walked for 5-min at 5 km h^−1^ before repeating the five-stage level-gradient IET to estimate speed and metabolic energy expenditure at VT_1_ using the methods described for visit 1 (Fig. 1; Supplementary Material S3). Participants then rested for 30-min before completing the exercise induced muscle damage indicators battery. Post-trial, participants recorded perceived leg muscle soreness at ~ 24-, ~ 48-, and ~ 72-h (Fig. 1).

Mathematically, loss of speed at VT_1_ can be attributed to: (i) reduced running economy at a given rate of metabolic energy expenditure (reduced energetic efficiency) and (ii) a reduction in the rate of metabolic energy expenditure at VT_1_ (reduced metabolic power). To determine the contributions made by changes in energetic efficiency and metabolic power to changes in speed at VT_1_ in LVL and DH, the rate of metabolic energy expenditure associated with VT_1_ in the POST IET was converted to speed using linear regression of the speed vs. energy expenditure relationship in the PRE IET (denoted POST_EE_PRE_Eff_). Therefore, POST_EE_PRE_Eff_ identifies the theoretical speed that the rate of whole-body metabolic energy expenditure measured at VT_1_ in POST would have produced with the same level of energetic efficiency as in the PRE IET. Accordingly, the contributions to changes in speed at VT_1_ made by changes in energetic efficiency and metabolic power, in LVL and DH, were calculated (Eq. 2). A detailed worked example of these calculations is available in Supplementary Material S4.

$$ {\text{Contribution of change in energetic efficiency to change in speed at the moderate-to-heavy intensity transition}}\, = \,{\text{POST}}{-}{\text{POST}}_{{{\text{EE}}}} {\text{PRE}}_{{{\text{Eff}}}} $$

2 $$ {\text{Contribution of change of metabolic energy expenditure to change in speed at the moderate-to-heavy intensity transition}}\, = \,{\text{POST}}_{{{\text{EE}}}} {\text{PRE}}_{{{\text{Eff}}}} {-}{\text{PRE}} $$

where energetic efficiency is equivalent to running economy, POST = speed at VT_1_ assessed in the post-exercise incremental exercise test, POST_EE_PRE_Eff_ = the speed that would have been achieved in the PRE assessment at the rate of metabolic energy expenditure observed at the moderate-to-heavy intensity transition in the POST assessment and PRE = speed at VT_1_ assessed in the pre-exercise incremental exercise test.

To quantify whole-body sweat loss in both trials, participants arrived in separate clothes from those worn during running. Pre- and post-exercise clothing mass, body mass, and water bottle mass were recorded. If a participant used the toilet during the trial, changes in body mass were recorded and the trial was ‘paused’ to maintain a total duration of 120-min. Dehydration was calculated by changes in body mass, adjusted for fluid consumption during the trial.

### Exercise-induced muscle damage indicators battery

Unilateral (right leg) maximal voluntary isometric knee extensor torque was measured using isokinetic dynamometry (Humac NORM, CSMi, Stoughton, MA, USA). Participants were seated (seat angle 80°), aligning the dynamometer’s axis of rotation with the lateral femoral epicondyle. The lever arm length was adjusted to position the pad at the distal tibia near the malleoli, ensuring full ankle mobility. The chest and right thigh were secured with straps to prevent extraneous movement. Positional measurements from visit 2 were replicated in visits 3 and 4. Torque measurements were taken at a knee joint angle of 80°, from full extension (180°), measured using goniometry with the dynamometer.

Participants completed a brief warm-up of 10 × 3-s contractions with a 5-s rest between contractions. For this, participants were instructed to “push at 50% effort”. After a 30-s rest, participants performed five maximal contractions, each lasting 3-s with a 30-s rest between contractions. To promote maximal force output, all participants were instructed to exert maximal force as fast as possible against the lever arm and to look at the monitor displaying force production (Baltzopoulos et al. 1991). Researchers gave a count of three before each push and provided strong verbal encouragement. The first two of the five contractions were omitted, and maximal voluntary isometric knee extensor torque was recorded as the greatest of the final three values.

Evidence of exercise-induced muscle damage was assessed by perceived leg muscle soreness before (PRE-LVL and PRE-DH), after (POST-LVL and POST-DH), and at ~ 24- (24H-LVL, 24H-DH), ~ 48- (48H-LVL, 48H-DH) and ~ 72-h (72H-LVL, 72H-DH) after the prolonged trial. The perceived leg muscle soreness was assessed using a visual analogue scale printed on a piece of white A4 paper, consisting of a 100 mm horizontal line anchored by two descriptors labelled from left (‘no soreness’) to right (‘worst soreness ever’). Participants squatted to parallel (~ 90° knee angle) and marked their perceived leg muscle soreness level on the printed horizontal line with a vertical line using a pen. Soreness was quantified by measuring the distance from the “no soreness” anchor point and the participant’s marked line (Davies et al. 2009, 2011). Assessments were conducted under researcher supervision for PRE-LVL, POST-LVL, PRE-DH, and POST-DH. For 24-, 48-, and 72-h measurements, participants used provided paper forms at home, sealing each in separate envelopes to avoid influencing subsequent responses.

### Statistical analysis

Data are presented as mean ± standard deviation unless otherwise specified. All analyses were performed in JASP (Version 0.18.3). Statistical significance was defined as *P* ≤ 0.05. The normality of datasets was assessed using the Shapiro–Wilk test. Two-way repeated measures analysis of variance was used to test the effect of time and condition on speed, metabolic energy expenditure and $\dot{V}O_{2}$ at VT_1_, maximal voluntary isometric knee extensor torque, perceived leg muscle soreness, and $\dot{V}O_{2}$, metabolic energy expenditure, carbohydrate oxidation, fat oxidation, heart rate and stride length during the 120-min prolonged run. If the assumption of sphericity was violated, as assessed by Mauchly's test of sphericity, the Greenhouse–Geisser correction was applied to adjust the degrees of freedom for the F-tests. When significant main effects were found, post-hoc analysis was conducted using paired *t*-tests with Holm-Bonferroni correction applied to adjust for multiple comparisons. One sample *t*-tests were used to determine whether changes in energetic efficiency and metabolic power were significant. Paired *t*-tests were used to compare total metabolic energy expenditure, oxygen consumption, carbohydrate oxidation, fat oxidation, sweat loss and dehydration between trials.

## Results

### Prolonged phase

Assessed in visits 1 and 2, ‘level’ (0% gradient) VT_1_ speed was 12.6 ± 0.8 km h^−1^ and ‘downhill’ (-10% gradient) VT_1_ speed was 15.8 ± 1.3 km h^−1^. Consequently, during the two-hour prolonged phase, level sections were completed at 11.3 ± 0.7 km h^−1^, and downhill sections at 14.3 ± 1.2 km h^−1^. Additionally, ‘level’ $\dot{V}O_{2}$ at VT_1_ was greater than ‘downhill’ (2.80 ± 0.45 L min^− 1^ vs. 2.46 ± 0.43 L min^−1^), as was metabolic energy expenditure (13.7 ± 2.3 kcal min^−1^ vs. 12.0 ± 2.1 kcal min^−1^) and heart rate (152 ± 9 b min^−1^ vs. 145 ± 12 b min^−1^).

**Fig. 2:**
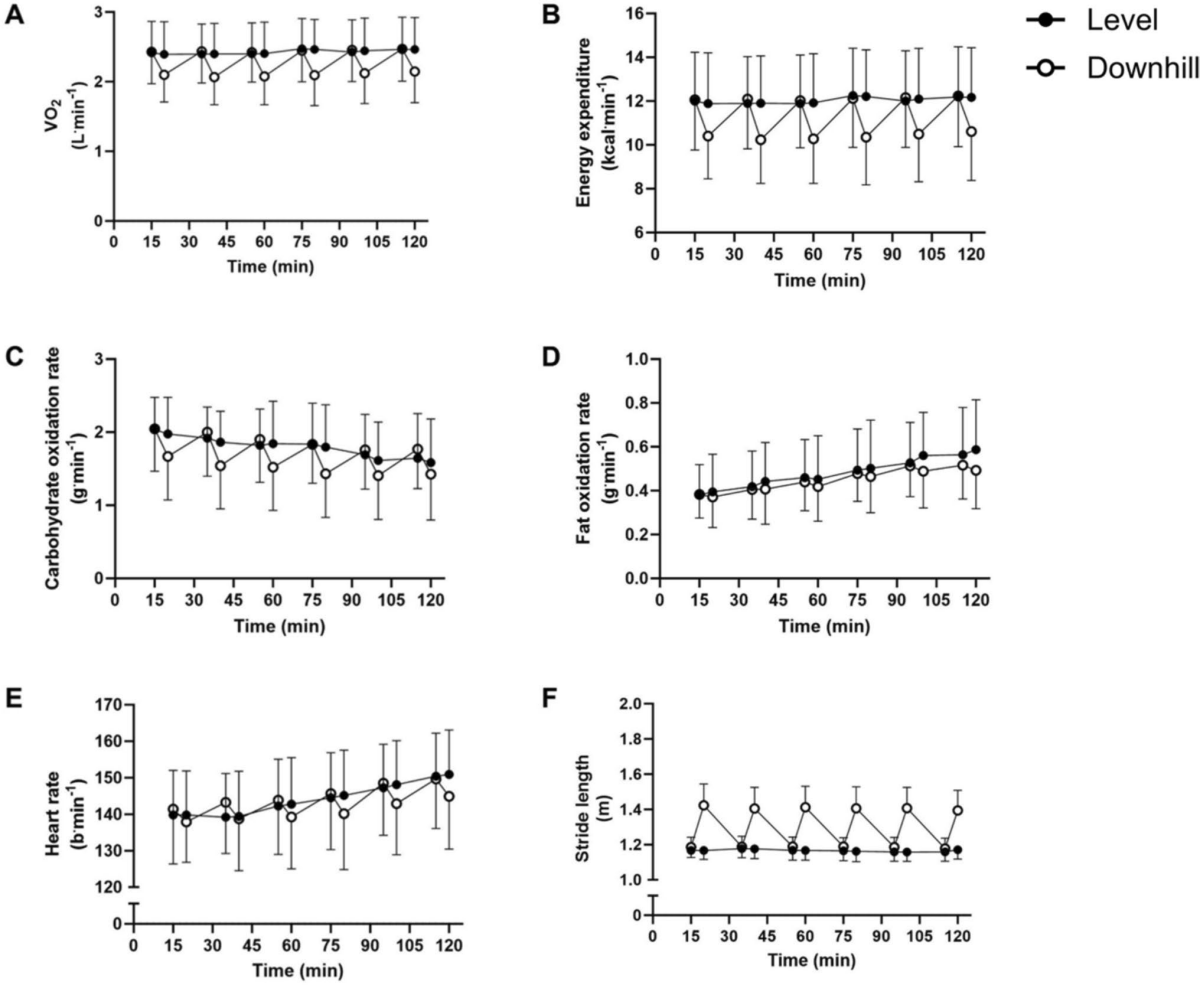
Physiological responses to prolonged running. **A** Rate of oxygen uptake ($\dot{V}O_{2}$ L min^−1^), **B** rate of metabolic energy expenditure (EE kcal min^−1^), **C** carbohydrate oxidation rate (g min^−1^), **D** fat oxidation rate (g min^−1^), **E** heart rate (b min^−1^) and **F** stride length (m) during the 120-min prolonged phase of the LVL and DH trials

There was a significant interaction between time and condition for metabolic energy expenditure and $\dot{V}O_{2}$ during the prolonged phase (*P* < 0.001; Figs. 2A and 4B). However, post-hoc analysis revealed no within- or between-condition effects when comparing 15-min values for metabolic energy expenditure and $\dot{V}O_{2}$ to all other level-gradient timepoints (all *P* > 0.05). Metabolic energy expenditure and $\dot{V}O_{2}$ were decreased in DH during the six downhill sections when compared with equivalent LVL timepoints (all *P* < 0.05). There was a significant time x condition interaction for carbohydrate (*P* < 0.001) and fat (*P* = 0.033) oxidation during the prolonged phase (Figs. 2C and 4D). Carbohydrate oxidation decreased significantly after 75-min and 95-min vs. 15-min in LVL and DH, respectively (all *P* < 0.01). Fat oxidation increased significantly after 80-min and 75-min vs. 15-min in LVL and DH, respectively (all *P* < 0.05). No significant between-condition differences were observed at each timepoint (all *P* > 0.05). Total metabolic energy expenditure over the 120-min was significantly greater in LVL compared to DH (1478 ± 267 kcal vs. 1403 ± 243 kcal, ∆ 5.5 ± 5.4%, *P* = 0.019). However, there was no significant difference in total oxygen uptake (286 ± 59 L vs. 275 ± 51 L, Δ 3.4 ± 5.7%, *P* = 0.053), carbohydrate oxidation (229 ± 59 g vs. 219 ± 62 g, ∆ 7.8 ± 21.9%, *P* = 0.432) or fat oxidation (56 ± 23 g vs. 52 ± 18 g, ∆ 10.8 ± 47.5%, *P* = 0.591) between trials. A significant interaction between time and condition was observed for heart rate (*P* = 0.029; Fig. 2E) during the prolonged phase, with significant increases after 100-min and 95-min vs. 15-min in LVL and DH, respectively (all *P* < 0.01). There were no significant between-condition differences at each timepoint (all *P* > 0.05).

A significant interaction between time and condition was observed for stride length (*P* < 0.001; Fig. 2F), but post-hoc analysis revealed no significant effect of time or condition on stride length in either trial when comparing 15-min values to other level-gradient timepoints (all *P* > 0.05). Stride length was shorter in DH during downhill sections compared to LVL (all *P* < 0.05).

### Moderate-to-heavy intensity transition

**Fig. 3:**
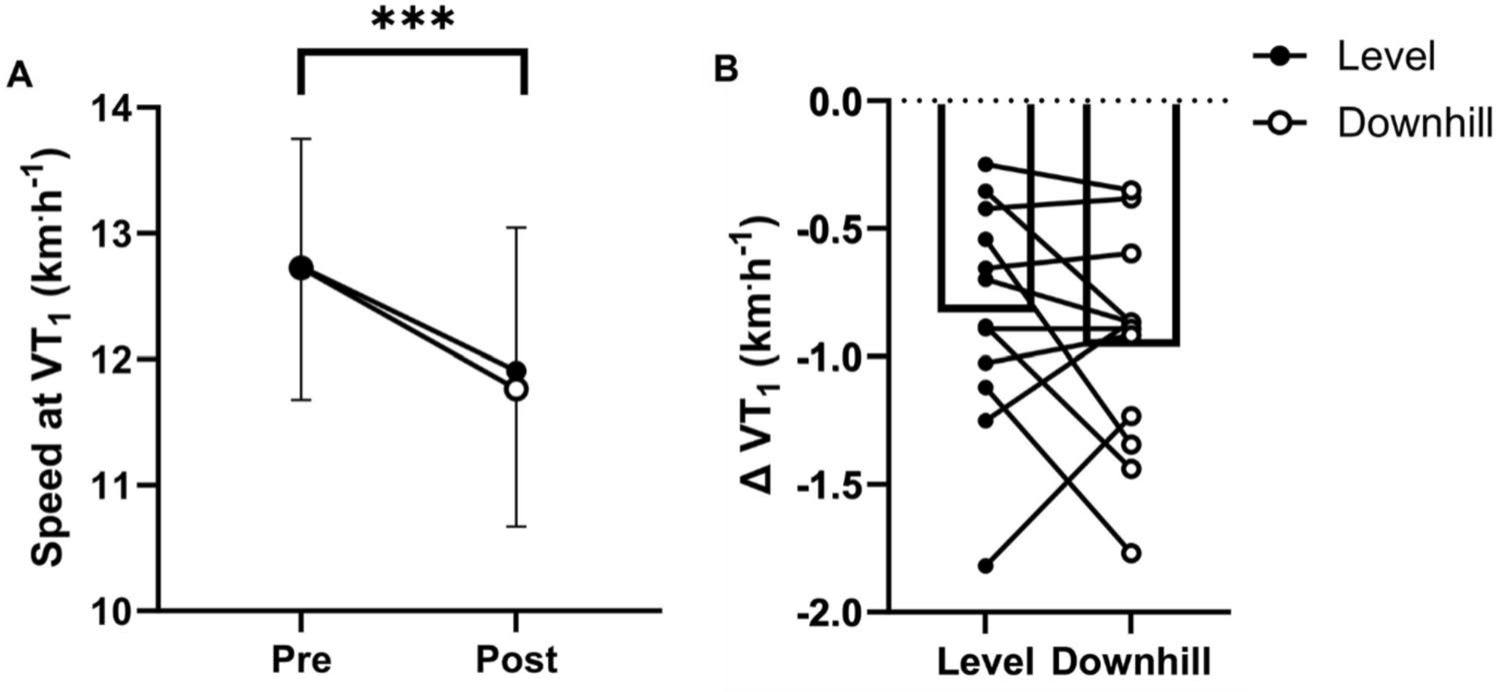
Speed at the moderate-to-heavy intensity transition. **A** Speed (km h^−1^) at the moderate-to-heavy intensity transition before (Pre) and after (Post) 120-min of prolonged running in the level and downhill trials, as determined by the first ventilatory threshold (VT_1_). Error bars indicate standard deviation. *** denotes *P* ≤ 0.001. **B** Change (∆) in speed (km h^−1^) at the moderate-to-heavy intensity before and after 120-min of prolonged running in the level and downhill trials. Bars indicate mean values while points with connecting lines indicate individual responses

PRE speed at VT_1_ was consistent (CV = 1.53%). There was a significant main effect of time (PRE vs. POST, P < 0.001), but no effect of condition (LVL vs. DH, *P* = 0.382) or time x condition interaction on speed at VT_1_. Specifically, speed at VT_1_ decreased from PRE to POST in both trials (12.7 ± 1.0 km h^−1^ to 11.9 ± 1.1 km h^−1^, ∆ − 6.2 ± 3.6% in LVL and 12.7 ± 1.0 km h^−1^ to 11.7 ± 1.1 km h^−1^, ∆ − 7.6 ± 3.2% in DH, Fig. 3). There was a significant main effect of time (PRE vs. POST, *P* < 0.001), but no effect of condition (LVL vs. DH, P < 0.386), on metabolic energy expenditure at VT_1_, which decreased PRE to POST in both trials (13.9 ± 2.3 kcal min^−1^ to 12.8 ± 2.1 kcal min^−1^, ∆ − 7.8 ± -1.1% in LVL and 13.7 ± 2.2 kcal min^−1^ to 12.7 ± 2.3 kcal min^−1^, ∆ − 7.1 ± − 1.0% in DH). There was a significant main effect of time (PRE vs. POST, P < 0.001), but no effect of condition (LVL vs. DH, P < 0.738), on $\dot{V}O_{2}$ at VT_1_, which decreased PRE to POST in both trials (2.75 ± 0.50 L min^−1^ to 12.8 ± 2.1 L min^−1^, ∆ − 5.4 ± − 3.8% in LVL and 2.76 ± 0.44 L min^−1^ to 2.56 ± 0.45 L min^−1^, ∆ − 7.5 ± 4.3 in DH).

**Fig. 4:**
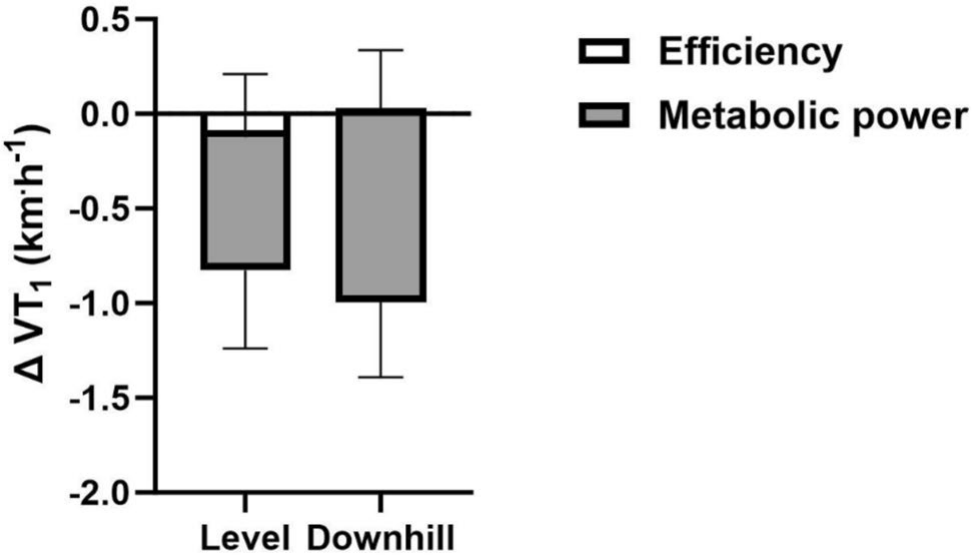
Physiological contributions to prolonged running-induced changes in the speed at the moderate-to-heavy intensity transition. The contributions of changes in energetic efficiency and metabolic power to prolonged running induced changes (∆) in the speed (km h^−1^) at the moderate-to-heavy intensity transition before and after 120-min of running in the level and downhill trials as determined by the first ventilatory threshold (VT_1_). Bars indicate mean values and error bars indicate standard deviation

Reduced metabolic power significantly contributed to the reduction in speed at VT_1_ in both trials (− 0.64 ± 0.38 km h^−1^, *P* < 0.001 in LVL and − 0.89 ± 0.29 km h^−1^, *P* < 0.001 in DH; Fig. 4). In contrast, there was no significant contribution of reduced energetic efficiency (− 0.12 ± 0.33 km h^−1^, *P* = 0.229 in LVL and − 0.02 ± 0.35 km h^−1^, *P* = 0.844 in DH; Fig. 4).

### Exercise-induced muscle damage indicators

**Fig. 5:**
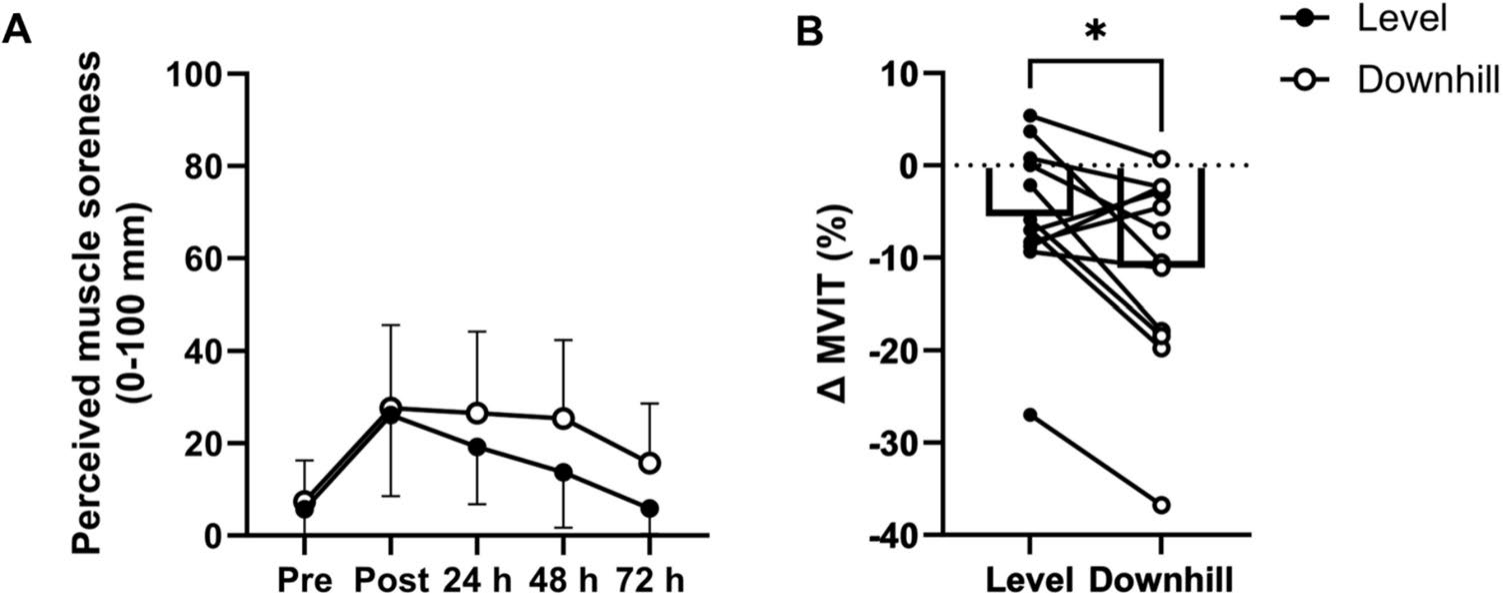
Muscle damage indicators. **A** Perceived leg muscle soreness (1–100 mm) estimated using a 100 mm visual analogue scale following a squat to parallel, before (Pre), 30 min after (Post), ~ 24 h, ~ 48 h and ~ 72 h after LVL and DH prolonged trials. Points indicate mean values and error bars indicate standard deviation. **B** Change (∆) in maximal voluntary isometric knee extensor torque (MVIT; %) before (PRE) and 30 min after (POST) LVL and DH trials. Bars indicate mean values while points with connecting lines indicate individual responses. *Denotes *P* ≤ 0.05

There was low variability in baseline perceived muscle soreness (IQR = 8.5 for LVL and 12 for DH). A significant main effect of time on perceived leg muscle soreness was observed (*P* = 0.004). At PRE, perceived leg muscle soreness was low (PRE-LVL, 6 ± 6/100 and PRE-DH, 8 ± 9/100, Fig. 5A). Perceived leg muscle soreness significantly increased at POST (POST-LVL, 26 ± 18/100 and POST-DH, 28 ± 19/100, *P* = 0.003) and remained increased at 24-h (24-LVL, 20 ± 13/100 and 24-DH, 27 ± 16/100, *P* = 0.013) and 48-h (48-LVL, 15 ± 12/100 and 48-DH, 27 ± 16, *P* = 0.02), before returning to PRE values at 72-h (72-LVL, 6 ± 6 /100 and 72-DH, 16 ± 13, *P* = 0.58). There was a significant main effect of condition on perceived leg muscle soreness (*P* = 0.029), but post-hoc analysis revealed no significant differences in perceived leg muscle soreness at each timepoint between conditions (all *P* > 0.05).

There was minimal variability in baseline maximal voluntary isometric peak torque (CV = 1.70%). A significant interaction between time and condition was found for maximal voluntary isometric knee extensor torque (P = 0.04, Fig. 5B). Post-hoc analysis showed no difference in baseline maximal voluntary isometric knee extensor torque (DH:224 224 ± 80 nM vs. 224 ± 79). However, torque significantly decreased from pre- to post-exercise DH (224 ± 80 nM vs. 201 ± 84 nM, ∆ − 11 ± 11%, P = 0.01), but not in LVL (224 ± 79 vs. 211 ± 78, ∆ − 6 ± 8%, P = 0.15).

### Sweat loss and dehydration

Sweat loss was significantly higher in DH compared with LVL (2.63 ± 0.60 L in LVL vs. 2.78 ± 0.59 in DH, ∆ 5.7%, *P* = 0.005), however, dehydration, as a percentage loss of BM was not significantly different between trials (2.58 ± 0.73% in LVL vs. 2.73 ± 0.62% in DH, P = 0.113).

## Discussion

We investigated the effect of prolonged running, and the effect of downhill running-induced eccentric load, on durability of the moderate-to-heavy intensity transition. Consistent with our hypothesis, speed at the moderate-to-heavy intensity transition decreased following 120-min of moderate-intensity running. This was explained by reduced metabolic power without changes in energetic efficiency. However, in contrast to our hypothesis, increasing the eccentric load did not appear to significantly exacerbate the decline in speed at the moderate-to-heavy intensity transition.

The observed decrease in speed at the moderate-to-heavy intensity transition following 120-min of moderate-intensity running in LVL and DH is consistent with previous findings in cycling (Stevenson et al. 2022; Gallo et al. 2024; Hamilton et al. 2024), and with a recent study in running (Nuuttila et al. 2024; Fig. 3). Metabolic power at the moderate-to-heavy intensity transition following prolonged running was reduced in both trials, as demonstrated by an ~ 7.8% and ~ 7.1% decrease in whole-body metabolic energy expenditure at the transition PRE-to-POST, similar to the ~ 6.3% reduction previously observed following prolonged cycling (Stevenson et al. 2022; Fig. 4). Mechanistically, this may be driven in part by exercise-induced muscle damage, which is associated with damage to contractile proteins (McCully 1986), disrupted myofilament structure (Morgan 1990; Talbot and Morgan 1996; Proske and Morgan 2001) and damage to the dystrophin complex (Gao and McNally 2015; Owens et al. 2019). Combined, this may impair the contractile ability of individual fibres via disruption of the contractile apparatus and increased membrane permeability which impairs excitation–contraction coupling (Macpherson et al. 1996). However, the absence of a condition effect on speed at the moderate-to-heavy intensity transition makes it difficult to discern an effect of exercise-induced muscle damage on shifts in this transition, as greater exercise-induced muscle damage in DH did not correspond with a larger decline in speed. The perceived leg muscle soreness increased significantly for 48-h following both trials, with greater effect in DH. However, the absence of time-point specific differences between LVL vs. DH suggests, while statistically detectable over the entire period, the greater effect in DH is relatively subtle and diffuse (Fig. 5A). Additionally, there was a significant reduction in maximal voluntary isometric knee extensor torque in DH of ~ 10%, but no significant decrease in LVL (Fig. 5B). These data suggest a muscle-damaging effect in both trials, with a more pronounced response in DH (Eston et al. 1996, 2000; Warren et al. 1999; Rowlands et al. 2001; Braun and Dutto 2003; Fig. 5). It is important to note that the ~ 5.5% reduction in overall energy cost during the prolonged phase of the DH trial may have influenced these results. Given the lower energy expenditure, the relative decline in VT_1_ speed in DH may have been greater. It also possible that glycogen depletion, which has been proposed as a mechanism of reduced metabolic power (Stevenson et al. 2022), contributed to impaired contractile function in individual muscle fibres following the prolonged run (Cairns and Renaud 2023; Ortenblad et al. 2013), and that preferential activation and therefore glycogen depletion in the most-aerobic, type I fibres shifted the pool of active fibres to a progressively less-oxidative phenotype (Nielsen et al. 2024). However, as we did not measure glycogen depletion in this study, we cannot further examine this hypothesis. Therefore, future studies assessing durability of the intensity domain transitions during running with measures of muscle glycogen are warranted.

We observed no reduction in energetic efficiency at the moderate-to-heavy intensity transition (Fig. 2), nor shifts in energy expenditure or $\dot{V}O_{2}$ during the prolonged run (Fig. 4), contrasting previous findings in both running (Unhjem 2024; Zanini et al. 2024) and cycling (Stevenson et al. 2022; Hamilton et al. 2024; Fig. 5). Studies that reported declines in running economy over 90- and 60-min regulated intensity at 100% LT_1_ (Zanini et al. 2024) and 70% $\dot{V}O_{2} \max$ speeds (Unhjem 2024), respectively, while we regulated intensity at 90% VT_1_ speed. A recent study reported reduced running economy following 90 min of running at 90% of speed at the moderate-to-heavy intensity transition, assessed using the lactate threshold in female runners, but not in males (Nuuttila et al. 2024). Regulating intensity at 100% LT_1_ may have placed participants in the heavy-intensity domain. This is due to the estimation involved in determining LT_1_ combined with the exercise-induced decline in speed at the moderate-to-heavy intensity transition observed in this study and others (Nuuttila et al. 2024). Additionally, due to the interindividual variability in fraction of $\dot{V}O_{2} \max$ at the moderate-to-heavy intensity transition, regulating intensity at 70% $\dot{V}O_{2} \max$ speed may have placed some participants in the heavy domain (Iannetta et al. 2020). Given that ~ 60% $\dot{V}O_{2} \max$ corresponded to the moderate-to-heavy intensity transition in the mentioned study (Unhjem 2024), many participants likely exercised in the heavy-intensity domain. Given distinct metabolic differences between exercise intensity domains (Black et al. 2017), this may explain discrepancies between our data and others, though reductions in energetic efficiency have been observed during moderate-intensity cycling (Stevenson et al. 2022; Hamilton et al. 2024). Higher-performing runners typically exhibit greater durability of running economy (Unhjem 2024; Zanini et al. 2024), but our participants were likely ‘lower performing’ than in the discussed studies, with $\dot{V}O_{2} {\text{peak}}$ values of ~ 51.5 ± 6.9 ml kg^−1^ min^−1^ in females and ~ 56.1 ± 3.5 ml kg^−1^ min^−1^ in males, compared to ~ 62.4 ml kg^−1^ min^−1^ (Zanini et al. 2024) and ~ 64.4 ml kg^−1^ min^−1^ (Unhjem 2024). The speed at the moderate-to-heavy intensity transition was also lower (13.2 km h^−1^ vs. 14.1 km h^−1^; Zanini et al. 2024). Therefore, despite indications of glycogen depletion, reflected by increased fat oxidation, and that this is associated with a shift from type I fibres to the less oxidative type II fibres (Nielsen et al. 2024), the absence of a significant change in energetic efficiency at the moderate-to-heavy intensity transition and energy expenditure and $\dot{V}O_{2}$ during prolonged exercise in this study remains unexplained and warrants further investigation.

Sweat loss and dehydration were assessed to rule out possible confounding effects of hydration status. Sweat loss was ~ 5.3% greater in DH vs. LVL; however, no significant differences in dehydration were observed between trials. This aligns with research indicating that heat production during eccentric exercise can exceed metabolic energy expenditure by up to three times (Nielsen 1966), leading to greater sweat loss during eccentric vs. concentric exercise (Nielsen 1966; Nielsen et al. 1972), likely because energy supplied by the treadmill is dissipated as heat during muscle lengthening (Davies and Barnes 1972). Participants consumed water ad libitum without specific hydration instructions, suggesting they intuitively increased water consumption during DH to compensate for the higher sweat loss. Thus, it is unlikely that increased sweat loss in DH substantially confounded our results.

Our data demonstrate that prolonged exercise induces a reduction in speed at the moderate-to-heavy intensity transition, a phenomenon now observed in both running and cycling (Stevenson et al. 2022; Gallo et al. 2024; Hamilton et al. 2024; Nuuttila et al. 2024; Fig. 5A and B). This highlights a critical issue: using assessments of speed from well-rested states to programme prolonged exercise may cause athletes to inadvertently drift from moderate to heavy intensity, increasing physiological stress and recovery time in training (Stanley et al. 2013), or negatively impacting performance in ultra-endurance running events performed largely at moderate-intensity (De Pauw et al. 2024). Additionally, training load models could consider the durability of the moderate-to-heavy intensity transition to enhance accuracy in quantifying training demands. Our findings also suggest that maintaining relative intensity across gradients results in similar degradation of the moderate-to-heavy intensity transition. This is challenging in real-world settings; runners often regulate intensity through heart rate and speed, which vary by gradient (Lemire et al. 2020). For example, in this study, speed at the moderate-to-heavy intensity transition was ~ 13.2 km h^−1^ on a 0% gradient and ~ 15.8 km h^−1^ on a − 10%, with heart rate ~ 152 b min^−1^ and ~ 145 b min^−1^, and metabolic energy expenditure ~ 13.7 kcal min^−1^ and ~ 12.0 kcal min^−1^, respectively. While grade-adjusted speed attempts to align energy cost across gradients (Minetti et al. 1994, 2002; Smyth and Muniz-Pumares 2020), it does not effectively regulate relative intensity, as metabolic energy expenditure relative to the moderate-to-heavy intensity transition decreases on negative gradients. Thus, using grade-adjusted speed on downhill sections to align energy cost of different gradients could push runners into a higher intensity domain than intended. Real-time power output measurement, common in cycling, may better regulate intensity across gradients if power output at intensity domain transition remains consistent. Given eccentric activity in downhill running produce greater force at a lower metabolic cost than concentric (Hill 1938; Bigland-Ritchie and Woods 1976; Aura and Komi 1986; Perrey et al. 2001), metabolic energy expenditure may be lower during downhill running despite equivalent power output. While running power meters are available, their reliability remains uncertain (Imbach et al. 2020; Cerezuela-Espejo et al. 2021; Linkis et al. 2021). Future research should explore whether power output at the transition is consistent across gradients and if power meters can provide reliable real-time data.

This study has several methodological considerations and limitations. Firstly, different IET protocols with varying stage durations were used, deliberately designed for specific objectives. The characterisation incremental trial used 3-min stages to initially estimate VT_1_ speed and $\dot{V}O_{2} {\text{peak}}$ using a standardised protocol in participants with diverse physiological profiles, providing a standardised basis for programming speeds during the prolonged run. Similarly, five 3-min downhill stages were used to estimate downhill-specific VT_1_, as longer downhill treadmill running became intolerable for some participants in pilot testing. This also meant that the protocol for setting level and downhill intensities in the prolonged trial was as similar as possible. The pre-/post-prolonged trials used 4-min stages to improve the precision of VT_1_ estimation, while the downhill test featured larger speed increments (2 km h^−1^ vs. 1 km h^−1^) to account for the greater range of speeds that can be achieved on a − 10% gradient. An important potential limitation is that VT_1_ speed estimated using the five-stage IETs relied on a limited number of data points, potentially reducing precision. A ramp test with more, shorter stages and smaller speed increments could improve sensitivity by capturing gas exchange data across a broader speed range. However, this would preclude steady-state gas exchange values required for calculating energetic efficiency and metabolic power. Thus, our protocol was designed to balance practical feasibility with physiological accuracy, though a ramp test may have provided better precision.

Beyond VT_1_ estimation, other methodological factors warrant consideration. Trials were matched for relative exercise intensity, resulting in different total energy costs between trials, as ‘downhill’ metabolic energy expenditure was lower than ‘level’ metabolic energy expenditure at respective VT_1_ speed (~ 13.7 kcal min^−1^ vs. 12.0 kcal min^−1^). This may have confounded the impact of exercise-induced muscle damage and/or glycogen depletion on the moderate-to-heavy intensity transition. Future work may match downhill and level protocols for energy cost, to better discern the effect of exercise-induced muscle damage on the moderate-to-heavy intensity transition. Logistical constraints limited maximal voluntary isometric knee extensor torque measurements to pre- and post-exercise. Despite a 30-min rest period following the running phase to allow for the dissipation of transient fatigue factors, the distinction between the contributions of acute fatigue factors and exercise-induced muscle damage to the reduction in peak torque is not entirely clear (Warren et al. 1999). Additionally, we did not measure levels of myofibrillar proteins in blood, another marker of exercise-induced muscle damage (Warren et al. 1999). Future studies should include measures of peak torque over several days post-exercise and myofibrillar protein analysis for improved exercise-induced muscle damage detection. Lastly, while greater maximal voluntary isometric knee extensor torque loss were observed in DH vs. LVL, this loss in torque (~ 6% in LVL and ~ 10% in DH) was less than the ~ 25% reported previously (Eston et al. 2000; Rowlands et al. 2001), potentially due to the steeper gradients and longer durations in those studies. Future research should consider steeper gradients and longer downhill durations to enhance exercise-induced muscle damage and better assess its impact on the moderate-to-heavy intensity transition.

To conclude, we demonstrated that prolonged moderate-intensity running significantly reduced speed at the moderate-to-heavy intensity transition, largely due to decreased metabolic power. Increasing the eccentric load during running at the same relative intensity did not influence the magnitude of the speed decline at this transition. Our data have implications for training programming, load monitoring, and pacing during ultra-endurance running events.

## Supplementary Information

Below is the link to the electronic supplementary material.

Supplementary file1 (DOCX 283 KB)

## Data Availability

Data is available from the corresponding author upon reasonable request.

## Abbreviations

DH: Downhill trial
IET: Incremental exercise test
LT_1_: First lactate threshold
LVL: Level trial
$\dot{V}O_{2}$: Rate of oxygen uptake
$\dot{V}O_{2} \max$: Maximal rate of oxygen uptake
$\dot{V}O_{2} {\text{peak}}$: Peak rate of oxygen uptake
VT_1_: First ventilatory threshold

## Code availability

None used.

## References

[CR1] Aura O, Komi PV (1986) The mechanical efficiency of locomotion in men and women with special emphasis on stretch-shortening cycle exercises. Eur J Appl Physiol Occup Physiol 55(1):37–43. 10.1007/BF00422890 3698985 10.1007/BF00422890

[CR2] Baltzopoulos V, Williams JG, Brodie DA (1991) Sources of error in isokinetic dynamometry: effects of visual feedback on maximum torque. J Orthop Sports Phys Ther 13(3):138–142. 10.2519/jospt.1991.13.3.138 18796847 10.2519/jospt.1991.13.3.138

[CR3] Bigland-Ritchie B, Woods JJ (1976) Integrated electromyogram and oxygen uptake during positive and negative work. J Physiol 260(2):267–277. 10.1113/jphysiol.1976.sp011515 978517 10.1113/jphysiol.1976.sp011515 PMC1309091

[CR4] Bijker KE, de Groot G, Hollander AP (2002) Differences in leg muscle activity during running and cycling in humans. Eur J Appl Physiol 87(6):556–561. 10.1007/s00421-002-0663-8 12355196 10.1007/s00421-002-0663-8

[CR5] Black MI, Jones AM, Blackwell JR, Bailey SJ, Wylie LJ, McDonagh ST, Thompson C, Kelly J, Sumners P, Mileva KN, Bowtell JL, Vanhatalo A (2017) Muscle metabolic and neuromuscular determinants of fatigue during cycling in different exercise intensity domains. J Appl Physiol 122(3):446–459. 10.1152/japplphysiol.00942.2016 28008101 10.1152/japplphysiol.00942.2016 PMC5429469

[CR6] Bontemps B, Vercruyssen F, Gruet M, Louis J (2020) Downhill running: what are the effects and how can we adapt? A narrative review. Sports Med 50(12):2083–2110. 10.1007/s40279-020-01355-z 33037592 10.1007/s40279-020-01355-z PMC7674385

[CR7] Braun WA, Dutto DJ (2003) The effects of a single bout of downhill running and ensuing delayed onset of muscle soreness on running economy performed 48 h later. Eur J Appl Physiol 90(1–2):29–34. 10.1007/s00421-003-0857-8 12783232 10.1007/s00421-003-0857-8

[CR8] Brownstein CG, Metra M, Sabater Pastor F, Faricier R, Millet GY (2022) Disparate mechanisms of fatigability in response to prolonged running versus cycling of matched intensity and duration. Med Sci Sports Exerc 54(5):872–882. 10.1249/MSS.0000000000002863 35072662 10.1249/MSS.0000000000002863

[CR9] Burnley M, Vanhatalo A, Fulford J, Jones AM (2010) Similar metabolic perturbations during all-out and constant force exhaustive exercise in humans: a (31)P magnetic resonance spectroscopy study. Exp Physiol 95(7):798–807. 10.1113/expphysiol.2010.052688 20360422 10.1113/expphysiol.2010.052688

[CR10] Cairns SP, Renaud JM (2023) The potassium-glycogen interaction on force and excitability in mouse skeletal muscle: implications for fatigue. J Physiol 601(24):5669–5687. 10.1113/JP285129 37934587 10.1113/JP285129

[CR11] Cerezuela-Espejo V, Hernandez-Belmonte A, Courel-Ibanez J, Conesa-Ros E, Mora-Rodriguez R, Pallares JG (2021) Are we ready to measure running power? Repeatability and concurrent validity of five commercial technologies. Eur J Sport Sci 21(3):341–350. 10.1080/17461391.2020.1748117 32212955 10.1080/17461391.2020.1748117

[CR12] Clark IE, Vanhatalo A, Bailey SJ, Wylie LJ, Kirby BS, Wilkins BW, Jones AM (2018) Effects of two hours of heavy-intensity exercise on the power-duration relationship. Med Sci Sports Exerc 50(8):1658–1668. 10.1249/MSS.0000000000001601 29521722 10.1249/MSS.0000000000001601

[CR13] Clark IE, Vanhatalo A, Thompson C, Joseph C, Black MI, Blackwell JR, Wylie LJ, Tan R, Bailey SJ, Wilkins BW, Kirby BS, Jones AM (2019a) Dynamics of the power-duration relationship during prolonged endurance exercise and influence of carbohydrate ingestion. J Appl Physiol 127(3):726–736. 10.1152/japplphysiol.00207.2019 31295069 10.1152/japplphysiol.00207.2019

[CR14] Clark IE, Vanhatalo A, Thompson C, Wylie LJ, Bailey SJ, Kirby BS, Wilkins BW, Jones AM (2019b) Changes in the power-duration relationship following prolonged exercise: estimation using conventional and all-out protocols and relationship with muscle glycogen. Am J Physiol Regul Integr Comp Physiol 317(1):R59–R67. 10.1152/ajpregu.00031.2019 30995104 10.1152/ajpregu.00031.2019

[CR15] Davies CT, Barnes C (1972) Negative (eccentric) work. II. Physiological responses to walking uphill and downhill on a motor-driven treadmill. Ergonomics 15(2):121–131. 10.1080/00140137208924416 5036082 10.1080/00140137208924416

[CR16] Davies RC, Rowlands AV, Eston RG (2009) Effect of exercise-induced muscle damage on ventilatory and perceived exertion responses to moderate and severe intensity cycle exercise. Eur J Appl Physiol 107(1):11–19. 10.1007/s00421-009-1094-6 19499242 10.1007/s00421-009-1094-6

[CR17] Davies RC, Eston RG, Fulford J, Rowlands AV, Jones AM (2011) Muscle damage alters the metabolic response to dynamic exercise in humans: a 31P-MRS study. J Appl Physiol 111(3):782–790. 10.1152/japplphysiol.01021.2010 21719720 10.1152/japplphysiol.01021.2010

[CR18] De Pauw K, Ampe T, Arauz YLA, Galloo X, Buyse L, Olieslagers M, Demuyser T, Corluy H, Lamarti S, Provyn S, Jones AM, Meeusen R, Roelands B (2024) Backyard running: pushing the boundaries of human performance. Eur J Sport Sci 24(10):1432–1441. 10.1002/ejsc.12190 39276329 10.1002/ejsc.12190 PMC11451558

[CR19] Duhamel TA, Perco JG, Green HJ (2006) Manipulation of dietary carbohydrates after prolonged effort modifies muscle sarcoplasmic reticulum responses in exercising males. Am J Physiol Regul Integr Comp Physiol 291(4):R1100-1110. 10.1152/ajpregu.00858.2005 16690765 10.1152/ajpregu.00858.2005

[CR20] Eston RG, Finney S, Baker S, Baltzopoulos V (1996) Muscle tenderness and peak torque changes after downhill running following a prior bout of isokinetic eccentric exercise. J Sports Sci 14(4):291–299. 10.1080/02640419608727714 8887208 10.1080/02640419608727714

[CR21] Eston RG, Lemmey AB, McHugh P, Byrne C, Walsh SE (2000) Effect of stride length on symptoms of exercise-induced muscle damage during a repeated bout of downhill running. Scand J Med Sci Sports 10(4):199–204. 10.1034/j.1600-0838.2000.010004199.x 10898263 10.1034/j.1600-0838.2000.010004199.x

[CR22] Gallo G, Faelli EL, Ruggeri P, Filipas L, Codella R, Plews DJ, Maunder E (2024) Power output at the moderate-to-heavy intensity transition decreases in a non-linear fashion during prolonged exercise. Eur J Appl Physiol 124(8):2353–2364. 10.1007/s00421-024-05440-3 38483635 10.1007/s00421-024-05440-3 PMC11322563

[CR23] Gao QQ, McNally EM (2015) The dystrophin complex: structure, function, and implications for therapy. Compr Physiol 5(3):1223–1239. 10.1002/cphy.c140048 26140716 10.1002/cphy.c140048 PMC4767260

[CR24] Hamilton K, Kilding AE, Plews DJ, Mildenhall MJ, Waldron M, Charoensap T, Cox TH, Brick MJ, Leigh WB, Maunder E (2024) Durability of the moderate-to-heavy-intensity transition is related to the effects of prolonged exercise on severe-intensity performance. Eur J Appl Physiol. 10.1007/s00421-024-05459-6 38546844 10.1007/s00421-024-05459-6 PMC11322397

[CR25] Hargreaves M, McConell G, Proietto J (1995) Influence of muscle glycogen on glycogenolysis and glucose uptake during exercise in humans. J Appl Physiol 78(1):288–292. 10.1152/jappl.1995.78.1.288 7713825 10.1152/jappl.1995.78.1.288

[CR26] Hill AV (1938) The heat of shortening and the dynamic constants of muscle. Proc R Soc Lond Ser B - Biol Sci 126(843):136–195. 10.1098/rspb.1938.0050

[CR27] Iannetta D, Inglis EC, Mattu AT, Fontana FY, Pogliaghi S, Keir DA, Murias JM (2020) A critical evaluation of current methods for exercise prescription in women and men. Med Sci Sports Exerc 52(2):466–473. 10.1249/MSS.0000000000002147 31479001 10.1249/MSS.0000000000002147

[CR28] Imbach F, Candau R, Chailan R, Perrey S (2020) Validity of the stryd power meter in measuring running parameters at submaximal speeds. Sports (Basel). 10.3390/sports8070103 32698464 10.3390/sports8070103 PMC7404478

[CR29] Jeukendrup AE, Wallis GA (2005) Measurement of substrate oxidation during exercise by means of gas exchange measurements. Int J Sports Med 26(Suppl 1):S28-37. 10.1055/s-2004-830512 15702454 10.1055/s-2004-830512

[CR30] Jones AM (2023) The fourth dimension: physiological resilience as an independent determinant of endurance exercise performance. J Physiol. 10.1113/JP284205 37606604 10.1113/JP284205

[CR31] Jones AM, Wilkerson DP, DiMenna F, Fulford J, Poole DC (2008) Muscle metabolic responses to exercise above and below the “critical power” assessed using 31P-MRS. Am J Physiol Regul Integr Comp Physiol 294(2):R585-593. 10.1152/ajpregu.00731.2007 18056980 10.1152/ajpregu.00731.2007

[CR32] Jones AM, Grassi B, Christensen PM, Krustrup P, Bangsbo J, Poole DC (2011) Slow component of VO2 kinetics: mechanistic bases and practical applications. Med Sci Sports Exerc 43(11):2046–2062. 10.1249/MSS.0b013e31821fcfc1 21552162 10.1249/MSS.0b013e31821fcfc1

[CR33] Jones AM, Burnley M, Black MI, Poole DC, Vanhatalo A (2019) The maximal metabolic steady state: redefining the “gold standard.” Physiol Rep 7(10):e14098. 10.14814/phy2.14098 31124324 10.14814/phy2.14098 PMC6533178

[CR34] Lemire M, Hureau TJ, Remetter R, Geny B, Kouassi BYL, Lonsdorfer E, Isner-Horobeti ME, Favret F, Dufour SP (2020) Trail runners cannot reach VO2max during a maximal incremental downhill test. Med Sci Sports Exerc 52(5):1135–1143. 10.1249/MSS.0000000000002240 31815832 10.1249/MSS.0000000000002240

[CR35] Linkis JE, Bonne TC, Bejder J, Rasmussen EK, Breenfeldt Andersen A, Nordsborg NB (2021) Reliability and validity of the SHFT running power meter. Sensors (Basel). 10.3390/s21227516 34833596 10.3390/s21227516 PMC8623456

[CR36] Macpherson PC, Schork MA, Faulkner JA (1996) Contraction-induced injury to single fiber segments from fast and slow muscles of rats by single stretches. Am J Physiol 271(5 Pt 1):C1438-1446. 10.1152/ajpcell.1996.271.5.C1438 8944625 10.1152/ajpcell.1996.271.5.C1438

[CR37] Marchand I, Tarnopolsky M, Adamo KB, Bourgeois JM, Chorneyko K, Graham TE (2007) Quantitative assessment of human muscle glycogen granules size and number in subcellular locations during recovery from prolonged exercise. J Physiol 580(Pt. 2):617–628. 10.1113/jphysiol.2006.122457 17272352 10.1113/jphysiol.2006.122457 PMC2075564

[CR38] Marcora SM, Bosio A (2007) Effect of exercise-induced muscle damage on endurance running performance in humans. Scand J Med Sci Sports 17(6):662–671. 10.1111/j.1600-0838.2006.00627.x 17346288 10.1111/j.1600-0838.2006.00627.x

[CR39] Maunder E, Seiler S, Mildenhall MJ, Kilding AE, Plews DJ (2021) The importance of “durability” in the physiological profiling of endurance athletes. Sports Med 51(8):1619–1628. 10.1007/s40279-021-01459-0 33886100 10.1007/s40279-021-01459-0

[CR40] McCully KK, Faulkner JA (1986) Characteristics of lengthening contractions associated with injury to skeletal muscle fibers. J Appl Physiol 61(1):293–299. 10.1152/jappl.1986.61.1.293 3733615 10.1152/jappl.1986.61.1.293

[CR41] Minetti AE, Ardigo LP, Saibene F (1994) Mechanical determinants of the minimum energy cost of gradient running in humans. J Exp Biol 195:211–225. 10.1242/jeb.195.1.211 7964412 10.1242/jeb.195.1.211

[CR42] Minetti AE, Moia C, Roi GS, Susta D, Ferretti G (2002) Energy cost of walking and running at extreme uphill and downhill slopes. J Appl Physiol 93(3):1039–1046. 10.1152/japplphysiol.01177.2001 12183501 10.1152/japplphysiol.01177.2001

[CR43] Morgan DL (1990) New insights into the behavior of muscle during active lengthening. Biophys J 57(2):209–221. 10.1016/S0006-3495(90)82524-8 2317547 10.1016/S0006-3495(90)82524-8 PMC1280663

[CR44] Nielsen B (1966) Regulation of body temperature and heat dissipation at different levels of energy-and heat production in man. Acta Physiol Scand 68(2):215–227. 10.1111/j.1748-1716.1966.tb03420.x

[CR45] Nielsen B, Nielsen SL, Petersen FB (1972) Thermoregulation during positive and negative work at different environmental temperatures. Acta Physiol Scand 85(2):249–257. 10.1111/j.1748-1716.1972.tb05258.x 5049420 10.1111/j.1748-1716.1972.tb05258.x

[CR46] Nielsen J, Schroder HD, Rix CG, Ortenblad N (2009) Distinct effects of subcellular glycogen localization on tetanic relaxation time and endurance in mechanically skinned rat skeletal muscle fibres. J Physiol 587(Pt 14):3679–3690. 10.1113/jphysiol.2009.174862 19470780 10.1113/jphysiol.2009.174862 PMC2742290

[CR47] Nielsen J, Holmberg HC, Schroder HD, Saltin B, Ortenblad N (2011) Human skeletal muscle glycogen utilization in exhaustive exercise: role of subcellular localization and fibre type. J Physiol 589(Pt 11):2871–2885. 10.1113/jphysiol.2010.204487 21486810 10.1113/jphysiol.2010.204487 PMC3112561

[CR48] Nielsen J, Jensen R, Ortenblad N (2024) Assessments of individual fiber glycogen and mitochondrial volume percentages reveal a graded reduction in muscle oxidative power during prolonged exhaustive exercise. Scand J Med Sci Sports 34(2):e14571. 10.1111/sms.14571 38389143 10.1111/sms.14571

[CR49] Nuuttila OP, Laatikainen-Raussi V, Vohlakari K, Laatikainen-Raussi I, Ihalainen JK (2024) Durability in recreational runners: effects of 90-min low-intensity exercise on the running speed at the lactate threshold. Eur J Appl Physiol. 10.1007/s00421-024-05631-y 39384626 10.1007/s00421-024-05631-y PMC11889008

[CR50] Ortenblad N, Nielsen J, Saltin B, Holmberg HC (2011) Role of glycogen availability in sarcoplasmic reticulum Ca2+ kinetics in human skeletal muscle. J Physiol 589(Pt 3):711–725. 10.1113/jphysiol.2010.195982 21135051 10.1113/jphysiol.2010.195982 PMC3055553

[CR51] Ortenblad N, Westerblad H, Nielsen J (2013) Muscle glycogen stores and fatigue. J Physiol 591(18):4405–4413. 10.1113/jphysiol.2013.251629 23652590 10.1113/jphysiol.2013.251629 PMC3784189

[CR52] Owens DJ, Twist C, Cobley JN, Howatson G, Close GL (2019) Exercise-induced muscle damage: what is it, what causes it and what are the nutritional solutions? Eur J Sport Sci 19(1):71–85. 10.1080/17461391.2018.1505957 30110239 10.1080/17461391.2018.1505957

[CR53] Perrey S, Betik A, Candau R, Rouillon JD, Hughson RL (2001) Comparison of oxygen uptake kinetics during concentric and eccentric cycle exercise. J Appl Physiol 91(5):2135–2142. 10.1152/jappl.2001.91.5.2135 11641354 10.1152/jappl.2001.91.5.2135

[CR54] Proske U, Morgan DL (2001) Muscle damage from eccentric exercise: mechanism, mechanical signs, adaptation and clinical applications. J Physiol 537(Pt 2):333–345. 10.1111/j.1469-7793.2001.00333.x 11731568 10.1111/j.1469-7793.2001.00333.x PMC2278966

[CR55] Rowlands AV, Eston RG, Tilzey C (2001) Effect of stride length manipulation on symptoms of exercise-induced muscle damage and the repeated bout effect. J Sports Sci 19(5):333–340. 10.1080/02640410152006108 11354612 10.1080/02640410152006108

[CR56] Smyth B, Muniz-Pumares D (2020) Calculation of critical speed from raw training data in recreational marathon runners. Med Sci Sports Exerc 52(12):2637–2645. 10.1249/MSS.0000000000002412 32472926 10.1249/MSS.0000000000002412 PMC7664951

[CR57] Stanley J, Peake JM, Buchheit M (2013) Cardiac parasympathetic reactivation following exercise: implications for training prescription. Sports Med 43(12):1259–1277. 10.1007/s40279-013-0083-4 23912805 10.1007/s40279-013-0083-4

[CR58] Stevenson JD, Kilding AE, Plews DJ, Maunder E (2022) Prolonged cycling reduces power output at the moderate-to-heavy intensity transition. Eur J Appl Physiol 122(12):2673–2682. 10.1007/s00421-022-05036-9 36127418 10.1007/s00421-022-05036-9 PMC9488873

[CR59] Swinnen W, Lievens E, Hoogkamer W, De Groote F, Derave W, Vanwanseele B (2024) Inter-individual variability in muscle fiber-type distribution affects running economy but not running gait at submaximal running speeds. Scand J Med Sci Sports 34(11):e14748. 10.1111/sms.14748 39461900 10.1111/sms.14748

[CR60] Talbot JA, Morgan DL (1996) Quantitative analysis of sarcomere non-uniformities in active muscle following a stretch. J Muscle Res Cell Motil 17(2):261–268. 10.1007/BF00124247 8793727 10.1007/BF00124247

[CR61] Tee JC, Bosch AN, Lambert MI (2007) Metabolic consequences of exercise-induced muscle damage. Sports Med 37(10):827–836. 10.2165/00007256-200737100-00001 17887809 10.2165/00007256-200737100-00001

[CR62] Unhjem RJ (2024) Changes in running economy and attainable maximal oxygen consumption in response to prolonged running: the impact of training status. Scand J Med Sci Sports 34(5):e14637. 10.1111/sms.14637 38671555 10.1111/sms.14637

[CR63] Warren GL, Lowe DA, Armstrong RB (1999) Measurement tools used in the study of eccentric contraction-induced injury. Sports Med 27(1):43–59. 10.2165/00007256-199927010-00004 10028132 10.2165/00007256-199927010-00004

[CR64] Zanini M, Folland JP, Blagrove RC (2024) Durability of running economy: differences between quantification methods and performance status in male runners. Med Sci Sports Exerc. 10.1249/MSS.0000000000003499 38857519 10.1249/MSS.0000000000003499

