# Supplementary material for: Prolonged running reduces speed at the moderate-to-heavy intensity transition without additional reductions due to increased eccentric load
Source: Eur J Appl Physiol. 2025 Apr 29;125(10):2897–910. doi: 10.1007/s00421-025-05792-4 (PMC12479608; doi:10.1007/s00421-025-05792-4)
Supplement: Supplementary file 1 — Supplementary file1 (DOCX 283 KB) [file 421_2025_5792_MOESM1_ESM.docx]

**Supplementary Material for**: Prolonged running reduces speed at the moderate-to-heavy intensity transition without additional reductions due to increased eccentric load

This supplementary material details the identification of speed at first ventilatory threshold (VT_1_) in the characterisation trial, downhill incremental trial, and PRE and POST incremental exercise tests during one of the prolonged trials. It also outlines the calculations used to determine the contributions of declines in running economy (energetic efficiency) and metabolic energy expenditure (metabolic power) to the decrease in speed at VT_1_ for one participant in this study.

**S1. Identification of level speed at VT_1_ from characterisation trial**

VT_1_ was identified as the breakpoint of the oxygen uptake (V̇O_2_) *vs*. ventilatory equivalent for oxygen (V̇E.V̇O_2_^-1^) relationship (Figure S1) and confirmed by the breakpoint in end-tidal oxygen partial pressure (Figure S2).


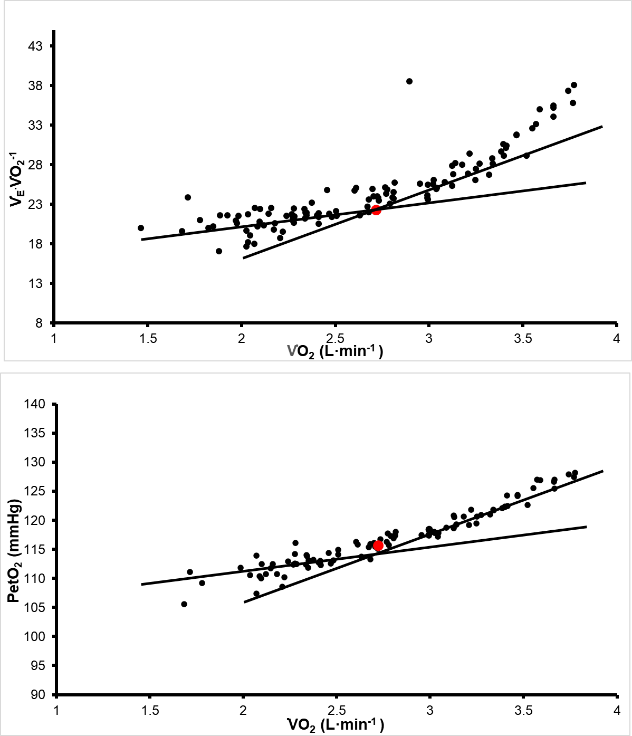


**Figure S1. Characterisation V̇O_2_ *vs.* V̇E.V̇O_2_^-1^** Data are 15s averages, V̇O_2_ (rate of oxygen uptake), V̇E.V̇O_2_^-1^ (ventilatory equivalent for oxygen), red point indicates V̇O_2_ at VT_1_.


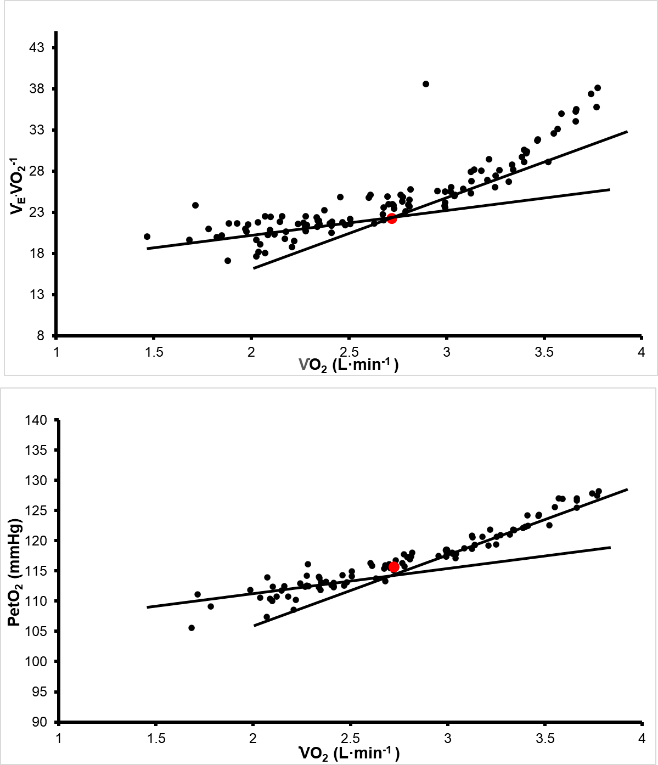


**Figure S2. Characterisation V̇O_2_ *vs.* PetCO_2_**  Data are 15s averages, V̇O_2_ (rate of oxygen uptake), PetCO_2_ (end-tidal oxygen partial pressure), red point indicates V̇O_2_ at VT_1_.

V̇O_2_ at level VT_1_ **~2.72 L·min^-1^**

V̇O_2_ at VT_1_ was then converted to speed by linear regression (Figure S3):


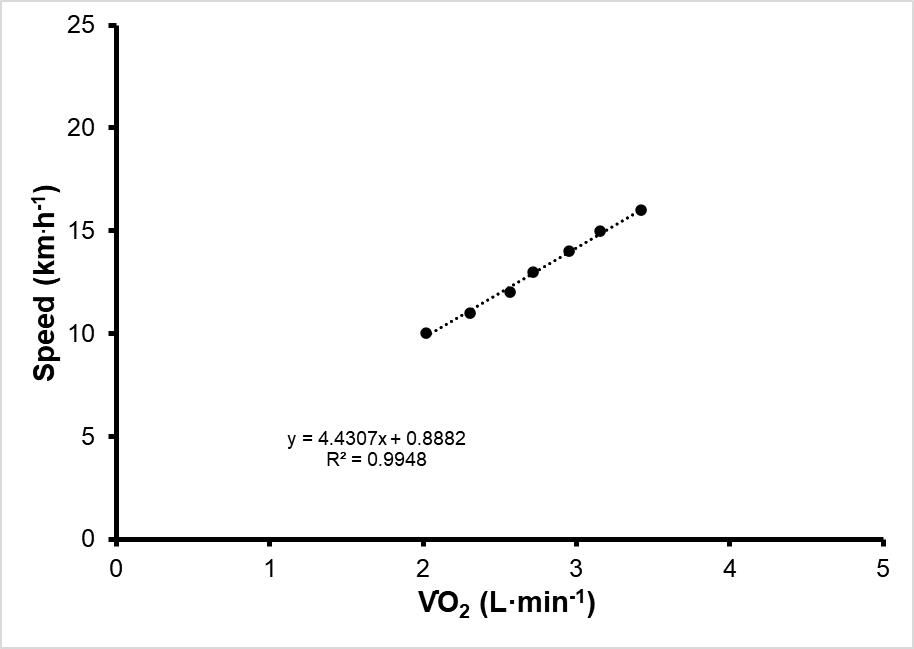


**Figure S3. Characterisation V̇O_2_ *vs.* speed** Data are 1 min averages from the final min of each stage, V̇O_2_ (rate of oxygen uptake).

$$\text{speed at V}\text{T}_{\text{1}}\text{=}\text{ }\text{4.4307×}\dot{\text{ }\text{V}}\text{O}_{\text{2}\text{ }}\text{at V}\text{T}_{\text{1}}\text{ }\text{+}\text{ }\text{0.8882}$$

$$s\text{peed at V}\text{T}_{\text{1}}\text{ }\text{=}\text{ }\text{4.4307}\text{ }\text{ ×}\text{ }\text{2.72}\text{ }\text{+}\text{ }\text{0.8882}$$

$$\text{speed at V}\text{T}_{\text{1}}\text{ }\text{=}\text{ }\text{12.9 km∙}\text{h}^{\text{-1}}$$

**S2. Identification of downhill speed at VT_1_ speed from downhill incremental trial**

VT_1_ was identified at the breakpoint of the V̇O_2_ vs. ventilatory equivalent for oxygen (V̇E.V̇O_2_^-1^) relationship (Figure S4) and confirmed by the breakpoint in end-tidal oxygen partial pressure (Figure S5).


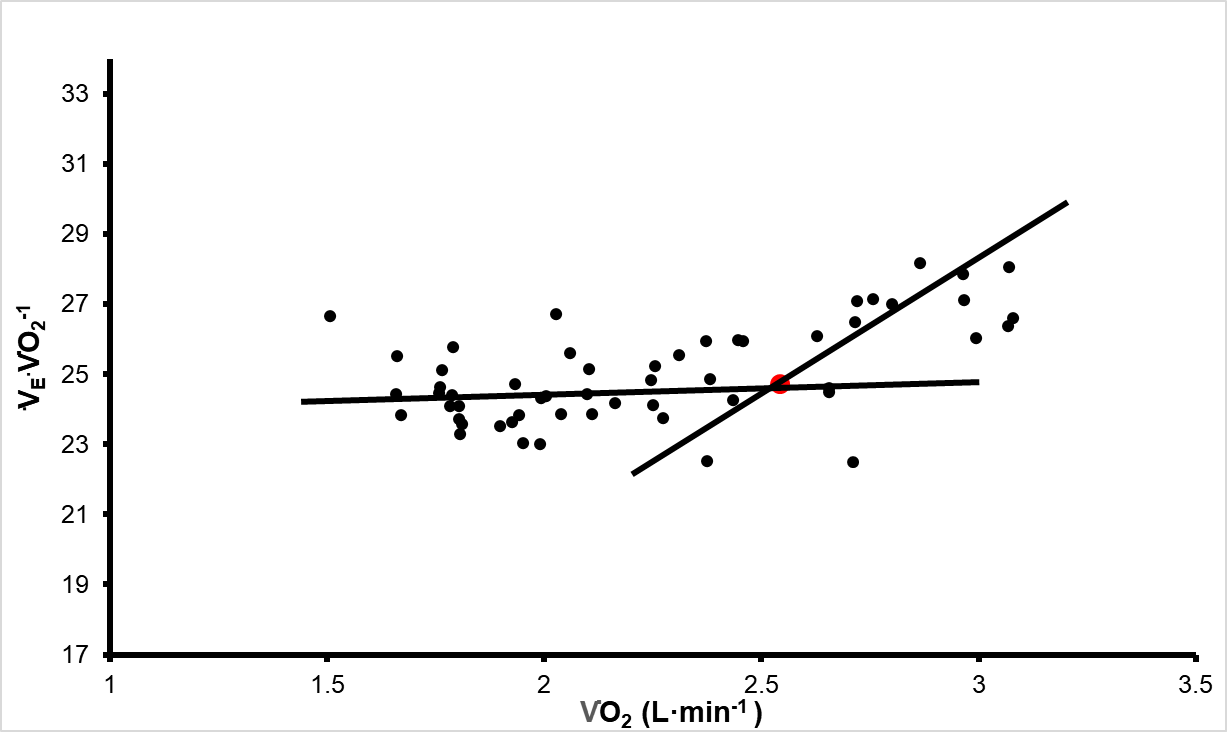


**Figure S4. Downhill V̇O_2_ *vs.* V̇E.V̇O_2_^-1^** Data are 15s averages, V̇O_2_ (rate of oxygen uptake), V̇E.V̇O_2_^- 1^ (ventilatory equivalent for oxygen), red point indicates V̇O_2_ at VT_1_.


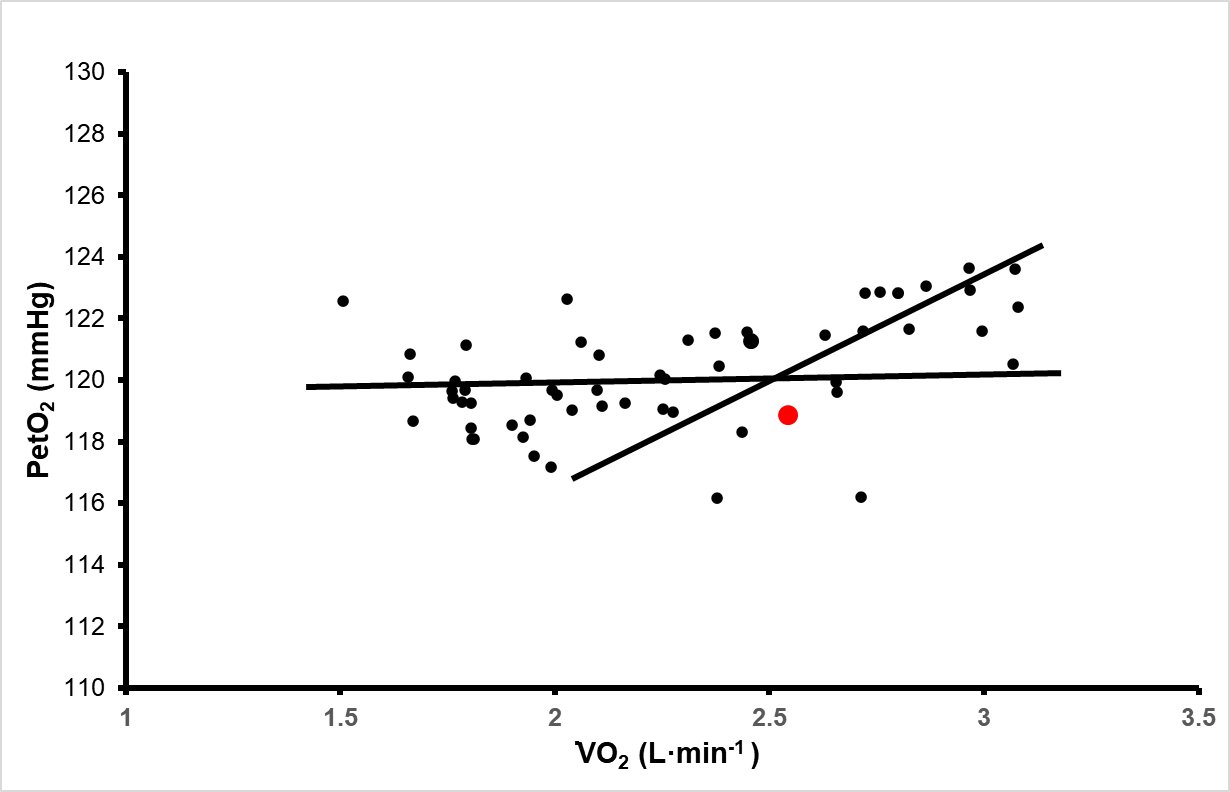


**Figure S5. Downhill V̇O_2_ *vs.* PetCO_2_**  Data are 15s averages, V̇O_2_ (rate of oxygen uptake), PetCO_2_ (end-tidal oxygen partial pressure), red point indicates V̇O_2_ at VT_1_.

V̇O_2_ at downhill VT_1_ ~**2.54 L·min^-1^**

V̇O_2_ at VT_1_ was then converted to speed by linear regression (Figure S6):

^
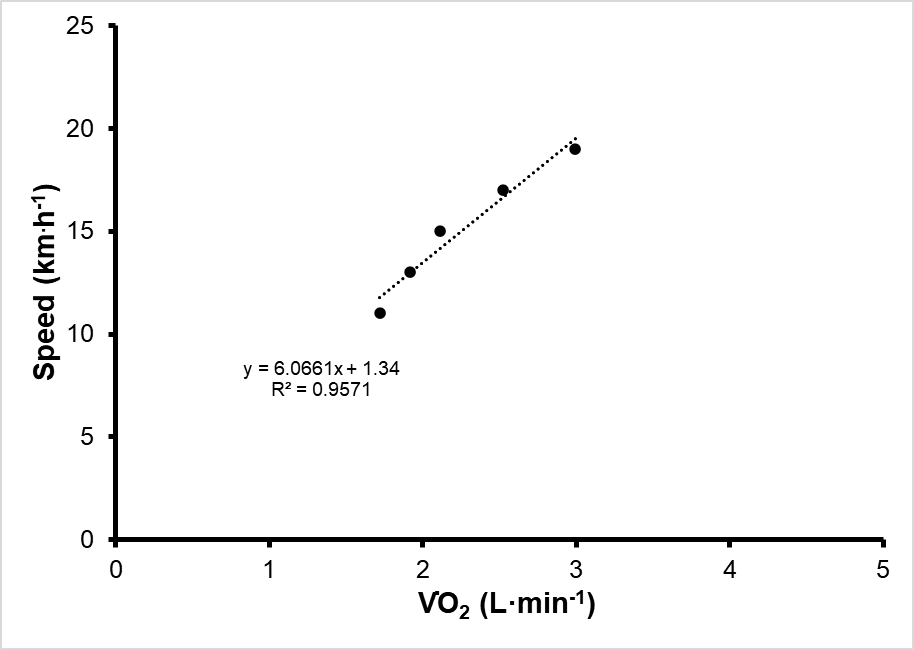
^

**Figure 6. Downhill V̇O_2_ *vs.* speed** Data are 1 min averages from the final min of each stage, V̇O_2_ (rate of oxygen uptake).

$$\text{speed at V}\text{T}_{\text{1}}\text{=}\text{ }\text{6.0661 ×}\dot{\text{ }\text{V}}\text{O}_{\text{2}}\text{ }\text{at V}\text{T}_{\text{1}}\text{ }\text{+}\text{ }\text{1.34}$$

$$\text{speed at V}\text{T}_{\text{1}}\text{=}\text{ }\text{6.0661 ×}\text{ }\text{2.54}\text{ }\text{+}\text{ }\text{1.34}$$

$$\text{speed at V}\text{T}_{\text{1}}\text{ }\text{=}\text{ }\text{16.7 km∙}\text{h}^{\text{-1}}$$

**S3. Identification of PRE and POST VT_1_ speed from 5-step incremental exercise test**

VT_1_ was identified as the breakpoint of the oxygen uptake (V̇O_2_) *vs*. ventilatory equivalent for oxygen (V̇E.V̇O_2_^-1^) relationship (Figure S7 and S9) and confirmed by the breakpoint in end-tidal oxygen partial pressure (Figure S8 and S10).

*PRE*

**
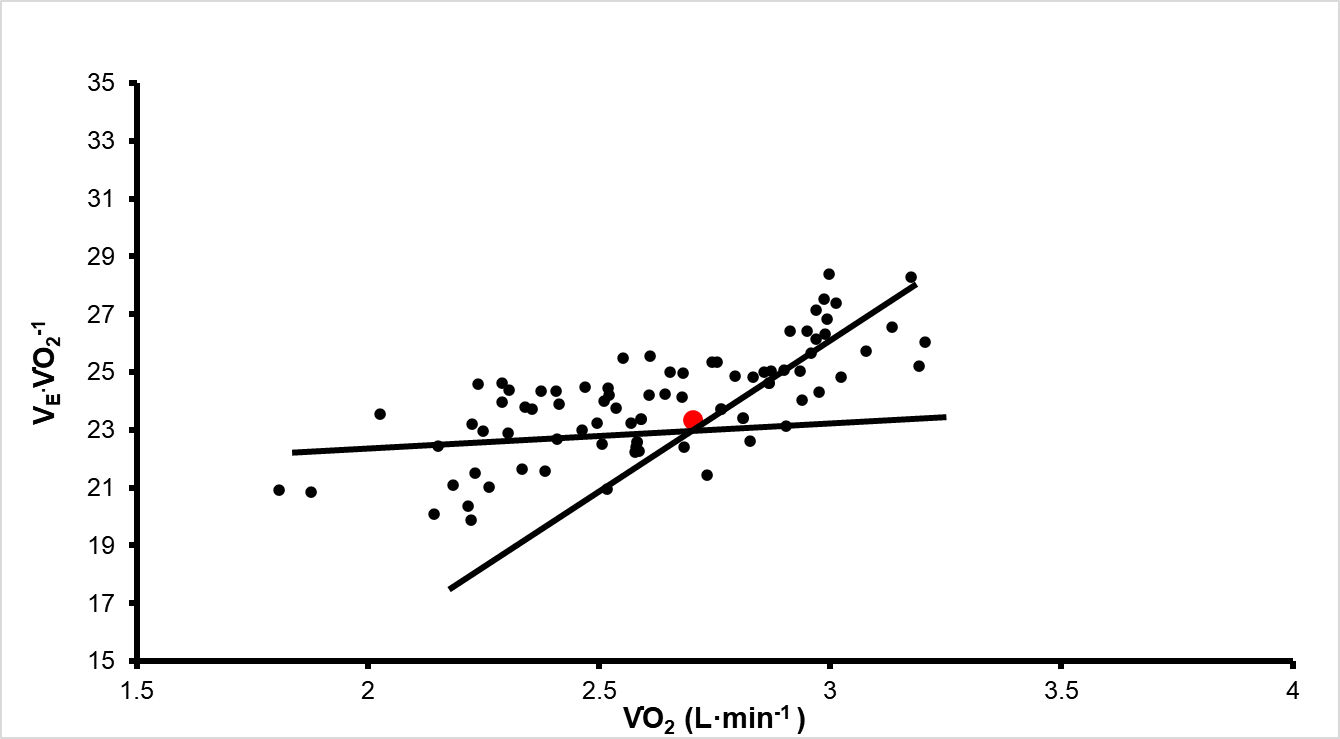
**

**Figure S7. PRE V̇O_2_ vs. V̇E^.^V̇O_2_^-1^** Data are 15s averages, V̇O_2_ (rate of oxygen uptake), V̇E.V̇O_2_^-1^ (ventilatory equivalent for oxygen), red point indicates V̇O_2_ at VT_1_.


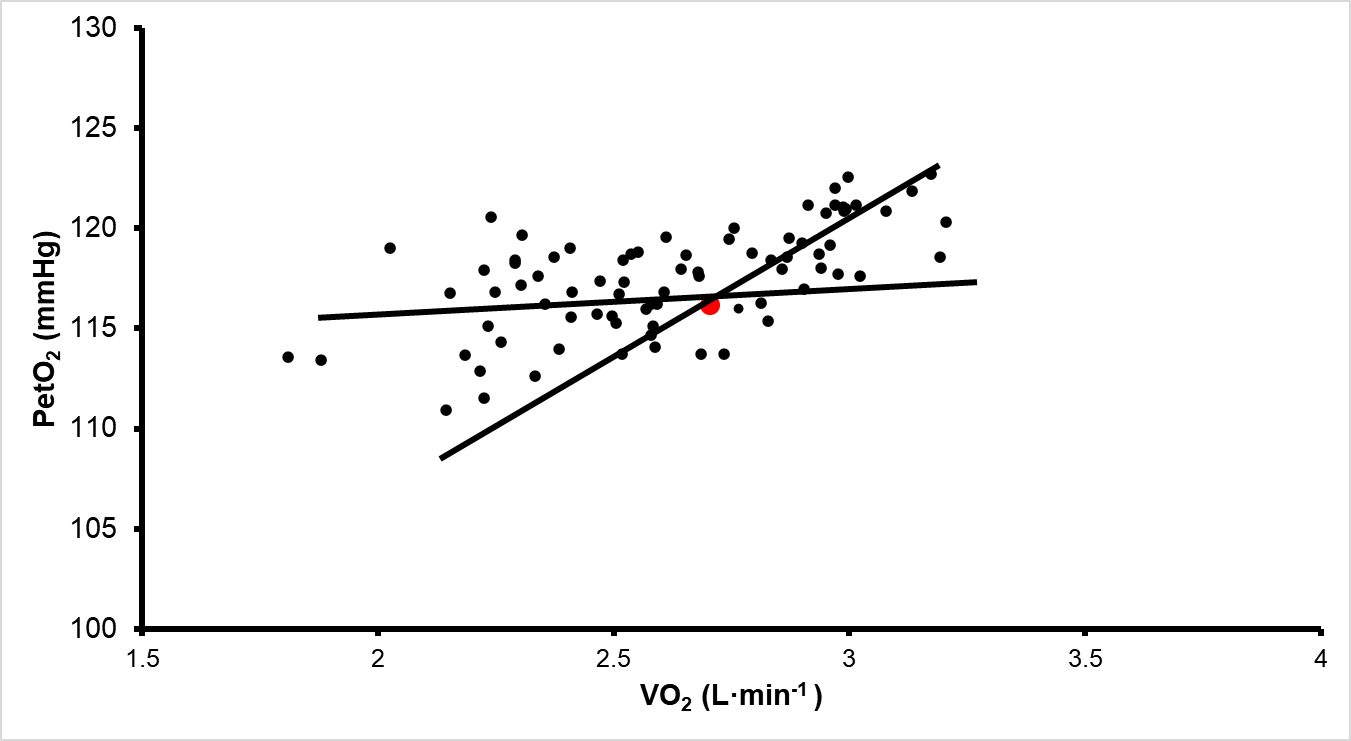


**Figure S8. PRE V̇O_2_ *vs.* PetCO_2_**  Data are 15s averages, V̇O_2_ (rate of oxygen uptake), PetCO_2_ (end-tidal oxygen partial pressure), red point indicates V̇O_2_ at VT_1_.

**PRE** V̇O_2_ at VT_1_ ~**2.70 L·min^-1^**

*POST*


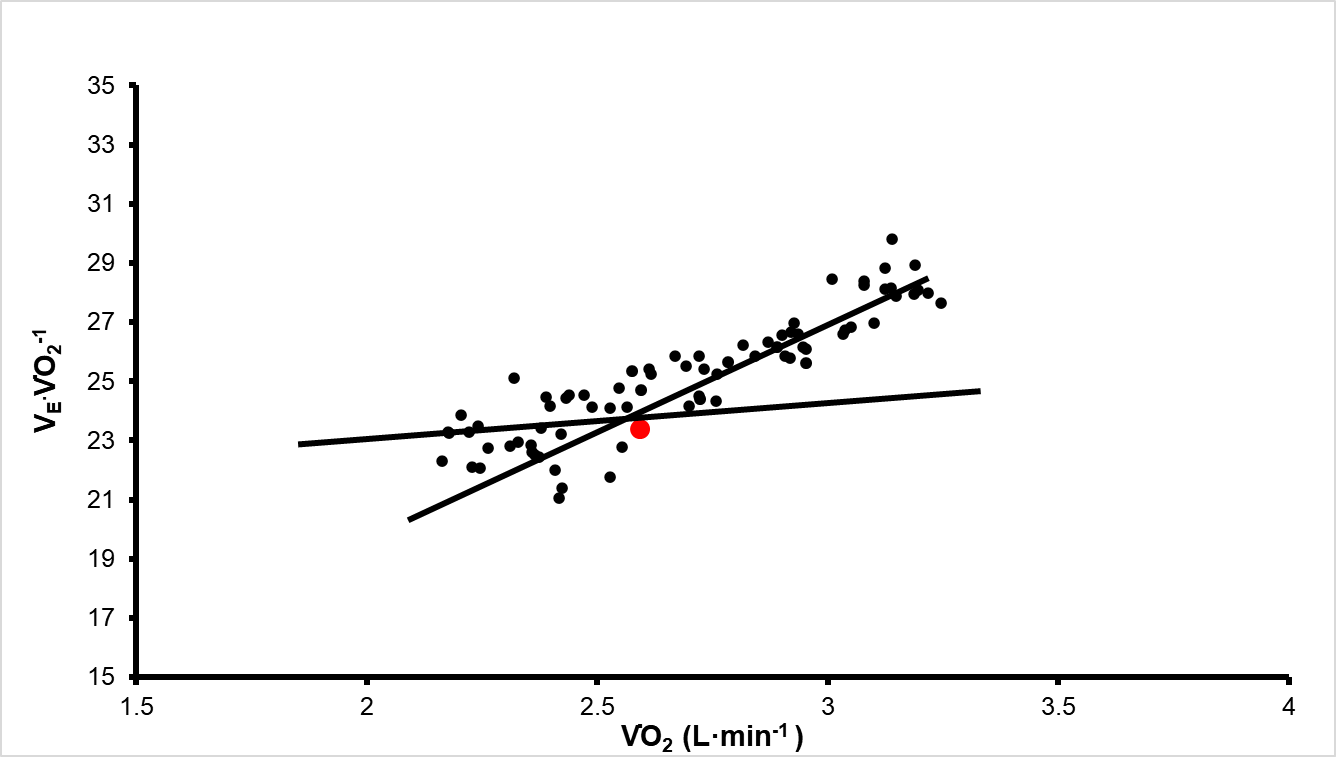


**Figure S9. POST V̇O_2_ vs. V̇E^.^V̇O_2_^-1^** Data are 15s averages, V̇O_2_ (rate of oxygen uptake), V̇E.V̇O_2_^-1^ (ventilatory equivalent for oxygen), red point indicates V̇O_2_ at VT_1_.


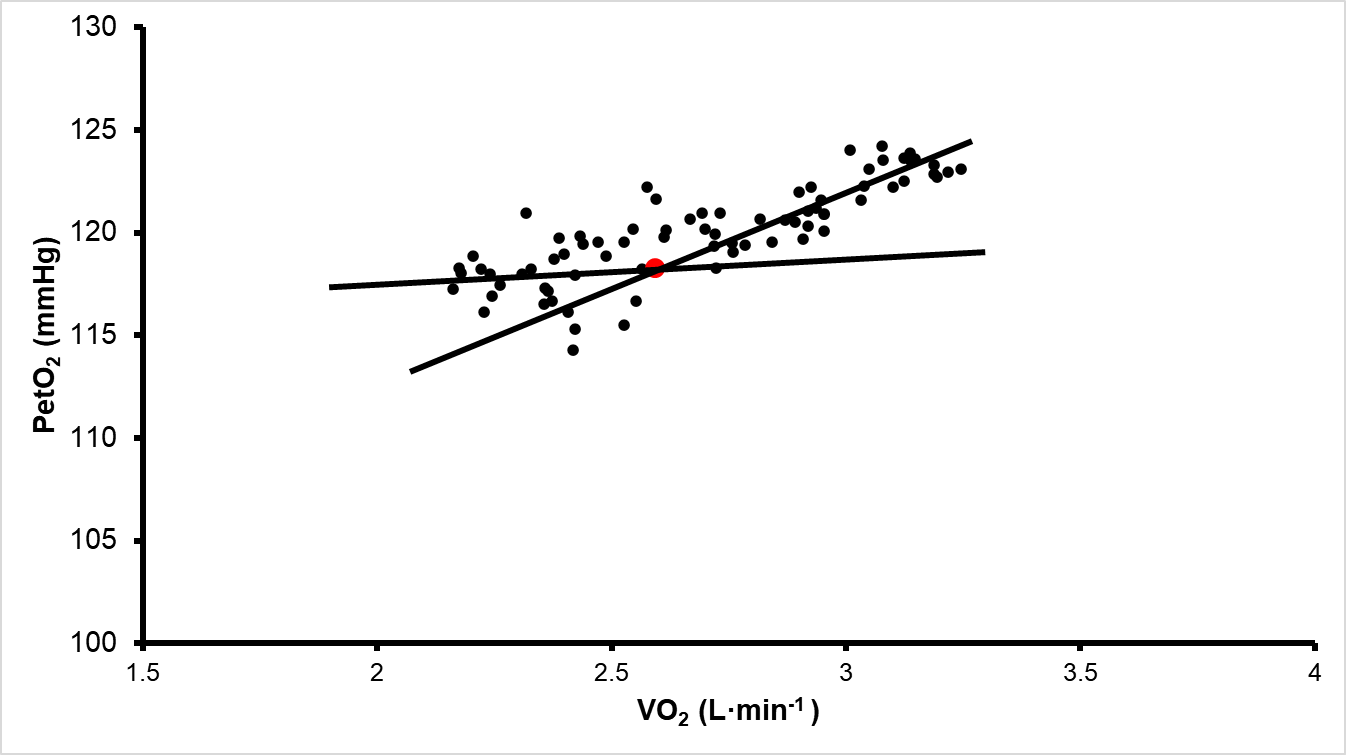


**Figure S10. POST V̇O_2_ *vs.* PetCO_2_**  Data are 15s averages, V̇O_2_ (rate of oxygen uptake), PetCO_2_ (end-tidal oxygen partial pressure), red point indicates V̇O_2_ at VT_1_.

**POST** V̇O_2_ at VT_1_ ~**2.59 L·min^-1^**

V̇O_2_ at VT_1_ was then converted to speed by linear regression (Figure S6)


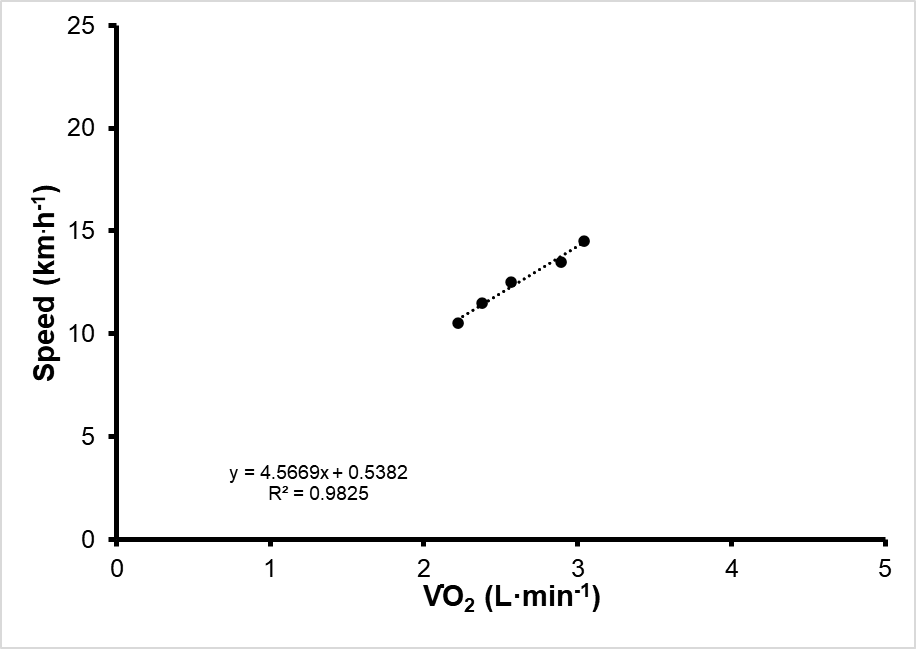


**Figure S11. PRE V̇O_2_ *vs.* speed** Data are 1 min averages from the final min of each stage, V̇O_2_ (rate of oxygen uptake).

$$\text{PRE}\text{ }\text{speed at V}\text{T}_{\text{1}}\text{=}\text{ }\text{4.5669 ×}\dot{\text{V}}\text{O}_{\text{2}\text{ }}\text{at V}\text{T}_{\text{1}}\text{ }\text{+}\text{ }\text{0.5382}$$

$$\text{PRE}\text{ }\text{speed at V}\text{T}_{\text{1}}\text{= 4.5669 × }\text{2.70 }\text{+ 0.5382}$$

**PRE** $\text{speed at V}\text{T}_{\text{1}}\text{ }\text{=}\text{ }\text{12.9 km∙}\text{h}^{\text{-1}}$

**
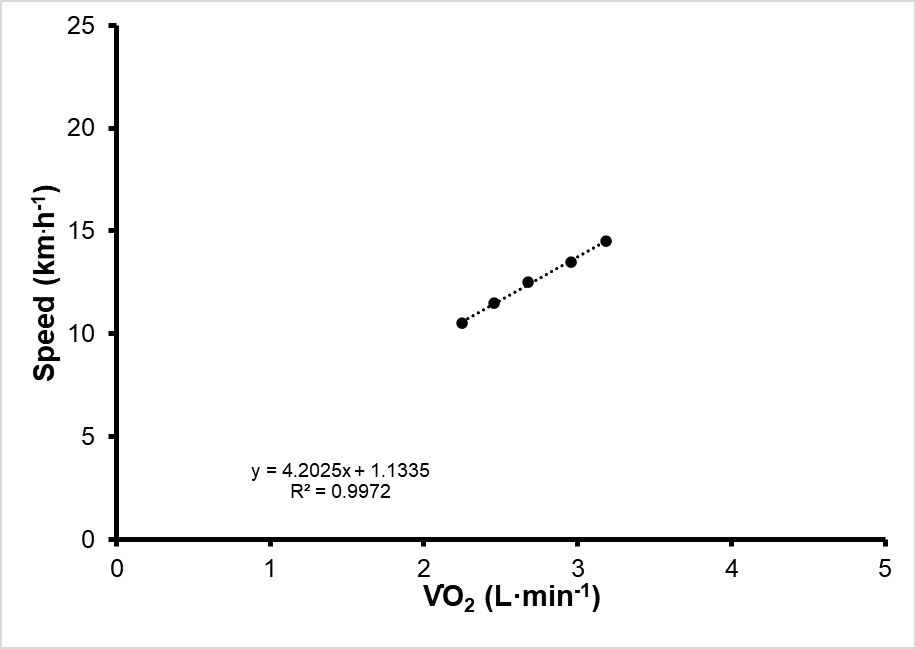
**

**Figure S12. POST V̇O_2_ *vs.* speed** Data are 1 min averages from the final min of each stage, V̇O_2_ (rate of oxygen uptake).

$$\text{POST }\text{speed at V}\text{T}_{\text{1}}\text{=4.2025 ×}\dot{\text{ V}}\text{O}_{\text{2 }}\text{at V}\text{T}_{\text{1}}\text{+ 1.1335}$$

$$\text{POST}\text{ }\text{speed at V}\text{T}_{\text{1}}\text{=}\text{ }\text{4.2025 ×}\text{ }\text{2.59}\text{ }\text{+}\text{ }\text{1.1335}$$

$$\text{POST}\text{ }\text{speed at V}\text{T}_{\text{1}}\text{=}\text{ }\text{12.0 km∙}\text{h}^{\text{-1}}\text{ }$$

**S4. Calculation of contributions of declines in metabolic power and energetic efficiency to decrease in speed at VT_1_**

This participant experienced a 0.9 km·h^-1^ decrease in speed at VT_1_. Mathematically, this speed increment can be attributed to:

- Reduced energetic efficiency (running economy): The speed achieved at a given metabolic energy expenditure is lower, meaning the participant had a greater metabolic energy per unit of speed in the POST trial *vs.* the PRE trial.
- Reduced metabolic power (metabolic energy expenditure): The absolute rate of energy expenditure at VT_1_ decreased from PRE to POST.

Below is a step-by-step breakdown of how these contributions are calculated:

*Step 1: Calculate the metabolic energy expenditure at VT_1_ in the POST trial*

Using the speed *vs*. metabolic energy expenditure relationship in the POST test, we first determine the metabolic energy expenditure (POST_EE_) at the POST VT_1_ speed (**12.0 km·h^-1^;** Figure S12):


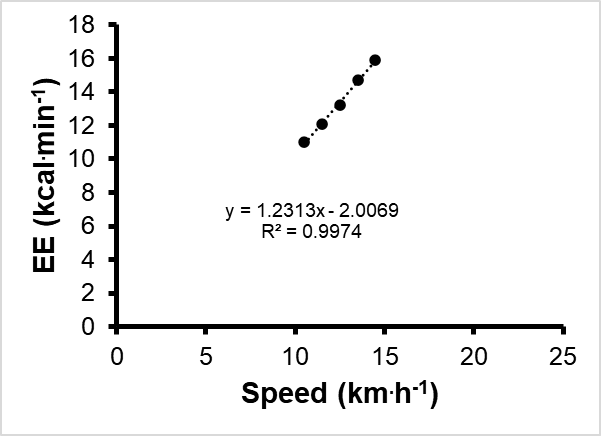


**Figure S12. POST speed *vs.* EE** Data are 1 min averages from the final min of each stage, EE (rate of metabolic energy expenditure).

POST_EE_ = 1.2313 x POST– -2.0069

POST_EE_ = 1.5313 x 12 – 2.0069

POST_EE_ = **12.7687 kcal^.^min^-1^**

*Step 2: Determine the theoretical PRE speed at the same energy expenditure (POST_EE_PRE_eff_)*

Next, we calculate the speed this metabolic energy expenditure at POST VT_1_ speed (**12.7687 kcal·min^-1^**) would have elicited in the PRE incremental exercise test using the energy expenditure *vs.* speed relationship from the PRE incremental exercise test (POST_EE_PRE_eff_; Figure S13):


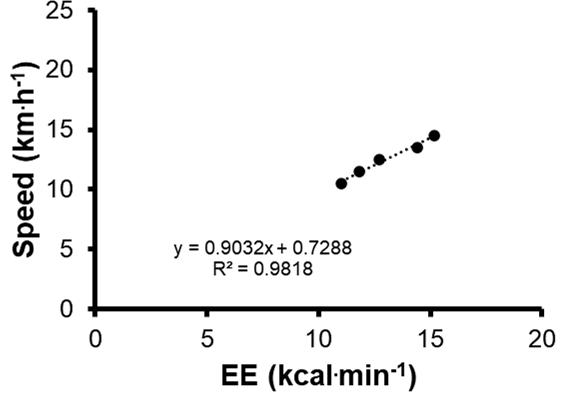


**Figure S13. PRE EE *vs.* speed** Data are 1 min averages from the final min of each stage, EE (rate of metabolic energy expenditure).

POST_EE_PRE_eff_ = 0.9032 x POST_EE_ + 0.7288

POST_EE_PRE_eff_ = 0.9032 x 12.7687 + 0.7288

POST_EE_PRE_eff_ = **12.3 km^.^h^-1^**

This means that, had the participant maintained their PRE running economy, they would have been running at ~**12.3 km·h^-1^** in the POST trial at a metabolic energy expenditure of ~**12.8 kcal^.^min^-1^**. However, their actual POST VT_1_ speed was ~**12.0 km·h^-1^,** indicating a decline in running economy.

The decrease in running economy is clear when the speed *vs.* metabolic energy expenditure relationship in both the PRE and POST incremental exercise trials are plotted together (Figure S14).


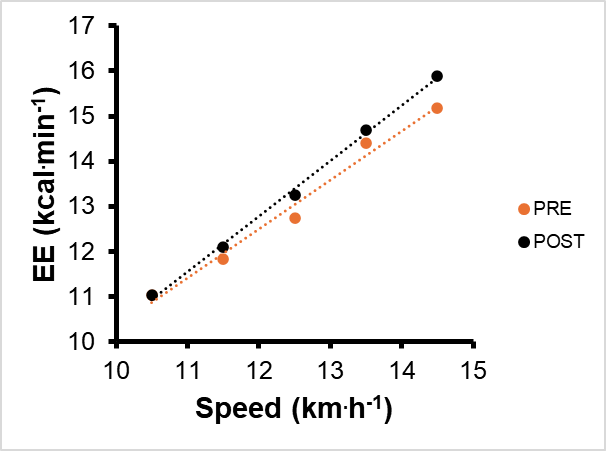


**Figure S15. PRE and POST speed *vs.* EE** Data are 1 min averages from the final min of each stage, EE (rate of metabolic energy expenditure). Not that energy expenditure per unit of speed is greater in the POST incremental exercise test *vs.* PRE.

*Step 3: Quantifying the contributions to the speed Ddecline*

The total loss in speed at VT_1_ was **0.9 km·h^-1^.** We now determine the relative contributions of energetic efficiency loss and metabolic power reduction.

Contribution of change in energetic efficiency to change in speed at the moderate-to-heavy intensity transition = POST - POST_EE_PRE_Eff_

= 12.0 - 12.3

= **-0.3 km^.^h^-1^**

*Interpretation:*

Had running economy remained the same, the participant would have been running **0.3 km·h^- 1^** faster at the same metabolic energy expenditure. Thus, **33.3%** of the speed decline was due to worsened running economy (Figure S16).

Contribution of change of metabolic energy expenditure to change in speed at the moderate-to-heavy intensity transition = POST_EE_PRE_Eff_ – PRE

= 12.3 - 12.9

= **-0.6 km^.^h^-1^**

*Interpretation*:

The remaining **0.6 km·h^-1^ (66.7%)** of the speed loss was due to a reduction in metabolic power at VT_1_ (Figure S16).


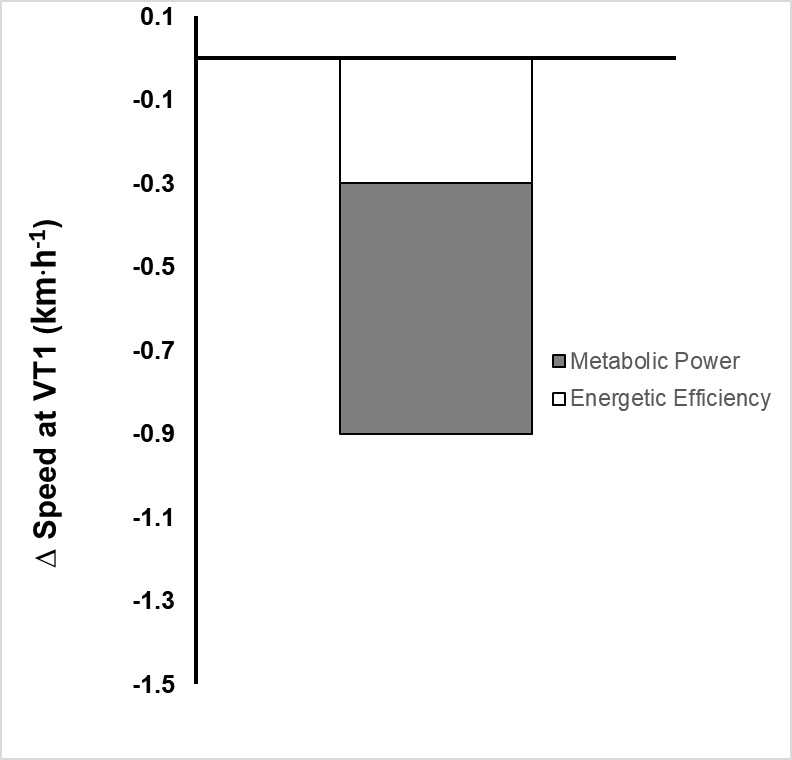


**Figure S16. Contribution of decreases in metabolic power and energetic efficiency to decrease in speed at VT_1_** Bars represent values for one individual participant.
